# Suppressor mutations reveal an NtrC-like response regulator, NmpR, for modulation of Type-IV Pili-dependent motility in *Myxococcus xanthus*

**DOI:** 10.1371/journal.pgen.1007714

**Published:** 2018-10-22

**Authors:** Daniel J. Bretl, Kayla M. Ladd, Samantha N. Atkinson, Susanne Müller, John R. Kirby

**Affiliations:** 1 Department of Microbiology and Immunology, Medical College of Wisconsin, Milwaukee, WI, United States of America; 2 Department of Biochemistry, University of Iowa, Iowa City, Iowa, United States of America; 3 Department of Bioinformatics, University of Iowa, Iowa City, Iowa, United States of America; U Alberta, UNITED STATES

## Abstract

Two-component signaling systems (TCS) regulate bacterial responses to environmental signals through the process of protein phosphorylation. Specifically, sensor histidine kinases (SK) recognize signals and propagate the response via phosphorylation of a cognate response regulator (RR) that functions to initiate transcription of specific genes. Signaling within a single TCS is remarkably specific and cross-talk between TCS is limited. However, regulation of the flow of information through complex signaling networks that include closely related TCS remains largely unknown. Additionally, many bacteria utilize multi-component signaling networks which provide additional genetic and biochemical interactions that must be regulated for signaling fidelity, input and output specificity, and phosphorylation kinetics. Here we describe the characterization of an NtrC-like RR that participates in regulation of Type-IV pilus-dependent motility of *Myxococcus xanthus* and is thus named NmpR, NtrC Modulator of Pili Regulator. A complex multi-component signaling system including NmpR was revealed by suppressor mutations that restored motility to cells lacking PilR, an evolutionarily conserved RR required for expression of *pilA* encoding the major Type-IV pilus monomer found in many bacterial species. The system contains at least four signaling proteins: a SK with a protoglobin sensor domain (NmpU), a hybrid SK (NmpS), a phospho-sink protein (NmpT), and an NtrC-like RR (NmpR). We demonstrate that Δ*pilR* bypass suppressor mutations affect regulation of the NmpRSTU multi-component system, such that NmpR activation is capable of restoring expression of *pilA* in the absence of PilR. Our findings indicate that pilus gene expression in *M*. *xanthus* is regulated by an extended network of TCS which interact to refine control of pilus function.

## Author summary

Sensing and responding to the environment are critical for bacterial survival and adaptation. Many bacteria sense environmental signals through biological machinery referred to as two-component signaling systems. These signaling systems are composed of a sensor histidine kinase and a cognate response regulator and serve to regulate gene expression in response to environmental cues. Due to their importance for survival, many bacterial genomes encode multiple two-component systems, some of which arose from gene duplication events. However, the mechanisms by which two-component systems integrate multiple inputs within larger networks remains poorly defined. In this study, we demonstrate that suppressor mutations within a *Myxococcus xanthus* signaling system of previously unknown function restore Type-IV pilus-dependent motility to cells lacking PilR, a response regulator typically required for production of the pilin monomer PilA. Suppressor mutations in Δ*pilR* cells led to activation of a related signaling system, resulting in expression of *pilA* and the corresponding production of PilA to restore motility. Thus, both PilSR and NmpSR coordinately regulate motility in *M*. *xanthus*. Collectively, our results expand our understanding of the activation of response regulators via phosphorylation and the mechanisms controlling multi-component signal transduction systems.

## Introduction

Two-component signaling systems (TCS) regulate numerous bacterial responses to environmental signals. These biological machines typically contain a membrane associated sensor histidine kinase (SK) and a cognate cytoplasmic response regulator (RR) that function together to propagate a response that is facilitated by the phosphorylation and dephosphorylation of the RR by the dual-function SK [1]. Following activation, RRs may function via different output domains, but the most common response is transcriptional regulation. Overall, TCS are critical for bacterial survival under varying environmental conditions and have well-established roles in metabolism, stress responses, virulence, motility, and many other physiological processes [2–9]. Despite the vast array of potential inputs and outputs, TCS have evolved to maintain remarkable specificity regarding phosphotransfer between cognate SK/RR pairs [10–12]. However, how TCS control the flow of information in the context of complex signaling networks within a given cell is less well-understood, especially in bacterial species with a high number of closely related TCS that have arisen from gene duplication.

One particularly important subfamily of RRs is known as the NtrC-like RRs, named after the nitrogen regulator NtrC of *Escherichia coli* and *Salmonella typhimurium* [13, 14]. This family of RRs is required for transcription initiation via interaction with RNA polymerase holoenzymes that specifically contain the σ^54^ sigma factor, which cannot initiate transcription on their own [15]. Unlike typical RRs, NtrC-like RRs contain a central ATPase domain that is required for hexamer or heptamer oligomerization [16–20] and is necessary for transcriptional activation by providing the energy for opening of the transcriptional bubble [21, 22]. Also, NtrC-like RRs may cause significant DNA bending, may function at relatively large distances (100–1000 bp) from transcriptional start points, and have been demonstrated to bind DNA elements both upstream and downstream of the start point. Thus, NtrC-like RRs are thought to function similarly to eukaryotic transcriptional machinery and are known as bacterial enhancer binding proteins [15, 23].

NtrC-like RRs are important transcription factors in numerous bacterial species, though many bacteria encode only a limited repertoire. For example, *E*. *coli* encodes only four (NtrC, ZraR, AtoC, and GlrR). However, some bacteria, especially environmental species with large genomes, encode an expanded number of NtrC-like RRs [24]. *Myxococcus xanthus*, a member of the δ-proteobacteria, encodes at least 27 *bona fide* NtrC-like RRs and a similar number of functionally related proteins that contain the σ^54^-interacting central ATPase domain but alternative sensing or output domains [25, 26]. It is believed many of the *M*. *xanthus* NtrC-like RRs arose via gene duplication and they are highly related at the nucleotide and amino acid level, making *M*. *xanthus* an ideal model organism to study how closely related signaling molecules maintain signaling fidelity or interact within complex networks [27]. Indeed, several NtrC-like RRs and related proteins collectively described as NtrC-like activators (Nla) are known to coordinate development and motility of *M*. *xanthus* [3, 25, 26, 28–36]. Consistent with the paradigm that TCS maintain signaling fidelity at the biochemical level, broad cross-phosphorylation ("cross talk") between these systems does not seem to occur in *M*. *xanthus* [12]. This suggests the NtrC-like pathways of *M*. *xanthus* interact to modulate behavior at other levels, such as integration of environmental signals or overlapping transcriptional outputs. Based on this view, we hypothesized that additional regulation of the expression of motility genes should exist for *M*. *xanthus* which depends largely on motility for its survival and coordination of complex social behaviors such as multi-cellular development and predation.

One method of *M*. *xanthus* motility is dependent on Type-IV pili (T4P). This motility is thought to be strictly dependent on PilR, the RR of the PilSR TCS in many bacteria including *M*. *xanthus* and *Pseudomonas aeruginosa* [5, 6, 26, 35, 37]. PilR is a member of the NtrC-like RR family and, under standard laboratory conditions, is necessary for transcription of *pilA* encoding the T4P monomer. In the absence of PilR, *M*. *xanthus* is unable to generate PilA and is therefore unable to move [26, 35]. However, in this study, we identify suppressor mutations in an *M*. *xanthus* Δ*pilR* strain that restore motility by activating a previously uncharacterized TCS. This system contains an NtrC-like RR (Mxan_4240) with no previously described function. We identified several independent mutations in *mxan_4240* that restore expression of *pilA*, and designate this gene *nmpR* (NtrC Modulator of Pili). Each mutation occurred in well-conserved domains within NmpR and likely affect activity by promoting or mimicking the phosphorylated RR state. Biochemical and genetic data also demonstrate that this NtrC-like RR is part of a complex signaling system that contains at least four components: an SK (Mxan_4246, NmpU), a potential phospho-sink protein with two isolated RR receiver domains (Mxan_4245, NmpT), a hybrid RR-SK (Mxan_4244, NmpS), and the output RR (Mxan_4240, NmpR).

## Results

### Identification of suppressor mutants that restore *M*. *xanthus* motility in the absence of PilR

**Fig 1:**
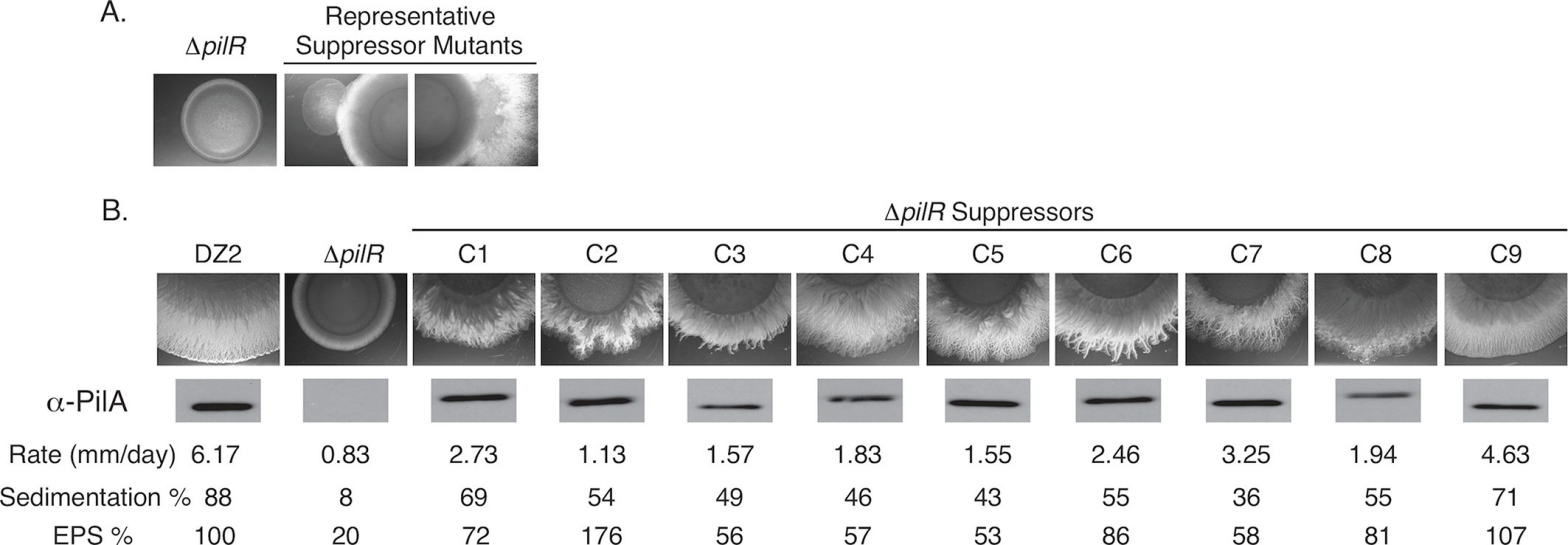
Characterization of *M*. *xanthus* Δ*pilR* suppressor mutants. (A) Representative suppressor mutations were observed after prolong incubation (7–14 days) on 0.5% agar. (B) Nine suppressor mutants were characterized for T4P-dependent motility related-phenotypes including motility rate (mm/day), sedimentation in static cultures reported as the percentage of lost absorbance at OD_600_ after 2 hours, and extracellular polysaccharide (EPS) production measured by the binding of Trypan blue to whole cells in solution and normalized to wild-type DZ2 which was set at 100%. All data represent the averages of at least 3 biological replicates. All suppressor mutant (C1-C9) measurements were significantly different (t-test, p<0.05) from wild-type DZ2 and Δ*pilR* with the exception of the EPS production of C6, C8, and C9 which were not different from that of the wild-type DZ2. Western blot analysis of total PilA from whole cell lysates was normalized to total protein determined by Bradford assay. All lysates were on the same blot, developed together, and can be directly compared to each other.

We have previously demonstrated that two RRs (PilR and PilR2) encoded within the *pil* locus of *M*. *xanthus* are independently necessary for T4P-dependent social motility [26]. In particular, the RR PilR (of the PilSR TCS) is necessary under standard laboratory conditions for expression of *pilA* encoding the major T4P subunit and is therefore essential for T4P-dependent motility [26, 35, 38]. *M*. *xanthus* T4P-dependent motility is assayed on 0.5% agar and is readily distinguished as radial expansion from an initial colony inoculum. Strains that are unable to move on this medium do not expand and have a smooth, delineated colony edge [39, 40]. During the course of our previous studies [26], we observed “flares” extending out from the edge of the *M*. *xanthus* Δ*pilR* colony indicating this otherwise non-motile strain acquired mutations allowing for restored motility (Fig 1A). Because of growth and colony motility rates, we typically assess *M*. *xanthus* T4P-dependent motility over the course of two days, but these flares only developed after prolonged incubation (7–14 days) at 32°C. Suppressor mutant cells were isolated from individual flares by transferring a ~1 cm^2^ area from the leading edge to fresh 0.5% agar. Colonies were allowed to move away from the transferred spot to limit any potential contamination of the original Δ*pilR* strain and then transferred to broth medium for preparation for long-term storage and future characterization. All isolated suppressor mutants were confirmed by PCR to have maintained their original background deletion of *pilR*. Independent cultures of the parental Δ*pilR* strain were then spotted twice more (total n = 3) and suppressor mutant flares were again observed with similar kinetics (i.e. 7–14 days). At the same time, the non-motile Δ*pilA* strain was assayed, and no suppressor mutant flares were observed, as expected. Therefore, suppressor mutations must have occurred in a gene distinct from *pilR*, yet require *pilA*, and thereby represent *pilR*-bypass suppressor mutations.

### Suppressor mutations restored production of exopolysaccharides, colony expansion, and sedimentation to the Δ*pilR* strain

To begin to determine the causative nature of restored motility in these suppressor strains, a subset of mutants was characterized for motility related phenotypes (Fig 1B). A total of nine mutant strains were assayed for three phenotypes associated with wild-type *M*. *xanthus* T4P-dependent social motility: 1) motility rates as measured by colony expansion over the course of five days; 2) the production of extracellular polysaccharides (EPS) that are essential for orienting cells within swarming colonies, stimulating T4P retraction, and regulating cell reversals [41, 42]; and 3) the percentage of cells that display sedimentation in standing culture due to the “stickiness” of both T4P and EPS (Fig 1B). All nine suppressor mutants displayed partial restoration of T4P-dependent motility, but at rates significantly lower than the wild-type cells. Similarly, all strains displayed varying levels of EPS production and sedimentation that were typically less than the wild-type but more than the Δ*pilR*. Collectively, these phenotypes strongly suggested that the restoration of motility to the suppressor mutant cells was due to their ability to produce T4P which requires production of the major pilin subunit, PilA. Indeed, anti-PilA immunoblot analyses of whole cell lysates of the nine suppressor mutants confirmed that they all produced near wild-type levels of PilA (Fig 1C). Because *pilA* gene expression could have resulted from mutations in its promoter region, we sequenced the *pilA* promoter (186 bp upstream of the translation start codon) in each of these strains. This analysis revealed no mutations had arisen in this known promoter region. Collectively, we concluded that restoration of PilA production, T4P-dependent motility, EPS production, and sedimentation of *M*. *xanthus ΔpilR* cells must have arisen at a unique locus relative to the *pilA* promoter.

### Whole genome sequencing revealed suppressor mutations localized within an uncharacterized TCS gene cluster

**Fig 2:**
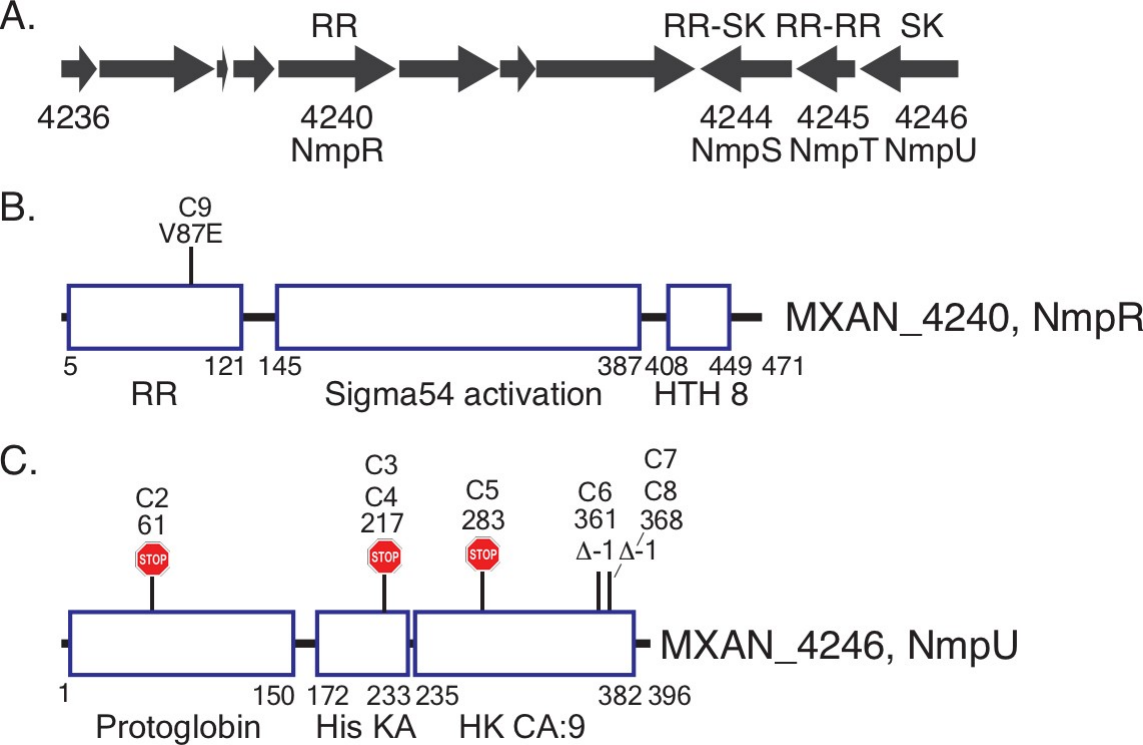
Suppressor mutations identified in uncharacterized TCS signaling genes. Suppressor strains were sequenced with the Illumina MiSeq platform and compared to the annotated *M*. *xanthus* DK1622 genome [27]. (A) Genomic organization of the locus containing the RR gene *mxan_4240* (*nmpR*) (B) and SK gene *mxan_4246* (*nmpU*) (C). This locus also contains a gene encoding a protein with two RR receiver domains (*mxan_4245*, *nmpT*, RR-RR) and an atypical hybrid response regulator/sensor kinase (*mxan_4244*, *nmpS*, RR-SK). The results of the mutations are depicted (B and C): a missense mutation in *nmpR* (V87E) and premature stop codons (stop signs) or frameshifts (Δ-1) in *nmpU*. The location of the mutations within each protein is to scale. The positions of each domain of these proteins is also indicated by amino acid number (RR = response regulator receiver domain, Sigma54 activation = central ATPase that interacts with σ^54^, HTH_8 = DNA-binding helix-turn-helix, Protoglobin = putative heme binding sensor domain, HisKA = domain of histidine phosphorylation and SK dimerization, HK CA:9 = ATPase. All domain nomenclatures are based on the Mist 2.2 database [87] and Pfam (http://pfam.xfam.org, [93]). The strain designation (C1-C9) is the same as in Table 1 and Fig 1.

To identify the location of the suppressor mutations, the nine characterized strains along with the wild-type *M*. *xanthus* DZ2 and Δ*pilR* parental strain were subjected to whole genome sequencing with the Illumina MiSeq platform. Raw sequences were assembled with SeqMan NGen (DNASTAR, Madison, WI) using the annotated *M*. *xanthus* DK1622 genome as a template [27]. The sequence data resulted in ~60x coverage for each genome. Importantly, the Δ*pilR* parental strain had no mutations relative to the laboratory wild-type *M*. *xanthus* DZ2 strain, and the *pilR* deletion was reconfirmed in each suppressor. Seven of the nine suppressor strains had an identifiable mutation (Table 1 and Fig 2) in *mxan_4246* (*nmpU*) encoding a SK and one strain had a mutation in *mxan_4240* (*nmpR*) encoding an NtrC-like RR. As the gene number indicates, these genes are in close proximity on the chromosome (Fig 2A). Mutations in the SK *nmpU* spanned the length of the gene and each resulted in either a premature stop codon or frame shift which we predict would result in truncated forms of the SK (Table 1 and Fig 2C). The single mutation identified in the RR *nmpR* was a missense mutation that changed a valine to glutamic acid at amino acid 87 (V87E) (Table 1, Fig 2B).

**Table 1:** *M*. *xanthus* Δ*pilR* suppressor mutations.

Strain	Mutation location^&^	Locus	Gene Characteristic^	Nucleotide change^+^	Amino acid change^$^
C1*	NA				
C2	2377162 5207011	*Intergenic* *mxan_4246*	SK	TT to CC C181T	Q61Stop
C3	5206543	*mxan_4246*	SK	C649T	Q217Stop
C4	5206543	*mxan_4246*	SK	C649T	Q217Stop
C5	5206345	*mxan_4246*	SK	C847T	Q283Stop
C6	5206106	*mxan_4246*	SK	Δ-1(1085)	FS-361
C7	5206087	*mxan_4246*	SK	Δ-1(1104)	FS-368
C8	5206087 7443662	*mxan_4246* *mxan_6013*	SK DnaJ/PilZ	Δ-1(1104) C601T	FS-368 P201S
C9	5199422	*mxan_4240*	Nla/RR	T260A	V87E

^&^, Mutation position based on *M*. *xanthus* DK1622 [27].

^, SK = sensor histidine kinase, Nla/RR (NtrC-like activator/Response regulator).

^+^, Nucleotide change listed as: consensus base/position in gene/suppressor mutation.

^$^, Amino acid change listed as: consensus amino acid/position in protein/amino acid change; Stop (stop codon), FS (frame shift).

*, NA = Not applicable, no mutation was identified.

### Response regulator *nmpR* has a gain-of-function mutation

**Fig 3:**
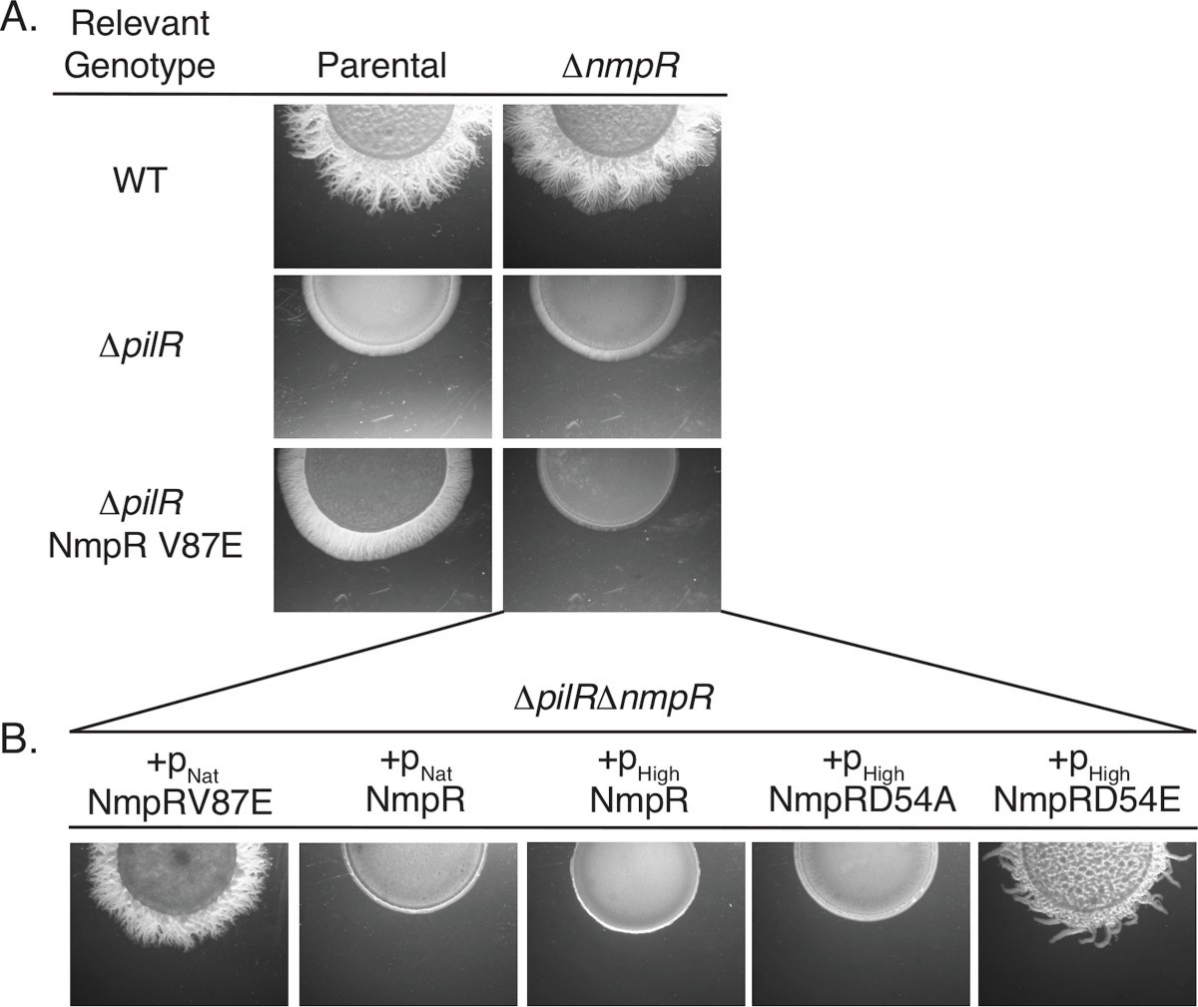
The NmpR V87E suppressor strain has a gain-of-function mutation. (A) In-frame *nmpR* deletions were constructed in the indicated strains and assayed for motility on 0.5% agar. All images depicted are after two days of motility. (B) Single copy complementation vectors were integrated at the Mx9 phage *attB* site and resulting strains assayed for motility. The p_Nat_ = 585 bp upstream of *mxan_4236* (See Fig 2A) and p_High_ = 623 bp upstream of *mxan_4894*, *groES* [43, 44] (See S1 Fig). D54A is an unphosphorylatable form of NmpR and D54E is a phosphomimetic.

Because the suppressors led to enhanced motility, we hypothesized that the amino acid change in the RR NmpR was the result of a gain-of-function mutation. To test this, we first constructed an in-frame *nmpR* deletion in the wild-type (*M*. *xanthus* DZ2), Δ*pilR*, and NmpRV87E strains. As expected, in the otherwise wild-type genetic background Δ*nmpR* cells remained motile (Fig 3A) since the native copy of *pilR* remained intact. In the absence of *pilR*, deletion of *nmpR* had no effect and this strain remained non-motile (Fig 3A). Finally, deletion of *nmpR*V87E in the suppressor strain resulted in a loss of motility (Fig 3A). Together these data strongly suggested that the NmpR V87E substitution confer a gain-of-function and confirmed this mutation is responsible for the restoration of motility for the Δ*pilR* cells. Several additional attempts to develop suppressor mutants in the Δ*pilR*Δ*nmpR* strain were unsuccessful despite several passages on motility agar plates, strongly suggesting that under the conditions tested NmpR is the only additional RR that can restore *pilA* expression and motility of the Δ*pilR* strain.

In order to determine if the NmpR V87E allele is sufficient to control expression of *pilA* and restore motility in the Δ*pilR*Δ*nmpR* mutant background, we generated a series of complementation constructs. Complementation of mutations was performed using expression constructs that are capable of integration in single copy at the *Mx9* phage attachment site (see Materials and Methods). Expression of a wild-type allele of *nmpR* from its putative native promoter (p_Nat_; 585 bp upstream of *mxan_4236*; Fig 2A) or from a high expression promoter (p_High_; 623 bp upstream of *mxan_4894*, *groES* [43, 44]; S1 Fig) was not able to restore motility to the Δ*pilR*Δ*nmpR* strain. In contrast, expression of *nmpR* V87E from its native promoter was sufficient to restore motility to Δ*pilR*Δ*nmpR* cells (Fig 3B). Because RRs typically require phosphorylation for activity, we expressed the phosphomimetic mutant form of NmpR (D54E) and a non-phosphorylatable variant (D54A). The D54E mutant form was sufficient to restore motility while the D54A mutant form was not (Fig 3B). Given that only the phosphomimetic version of NmpR (D54E) was capable of restoring motility, we hypothesize the suppressor variant (V87E) mimics or promotes an active conformation of this RR to restore *pilA* expression and motility to the Δ*pilR* strain.

### Identification of additional *nmpR* mutations that restore T4P-dependent motility in the *M*. *xanthus* Δ*pilR* background

**Fig 4:**
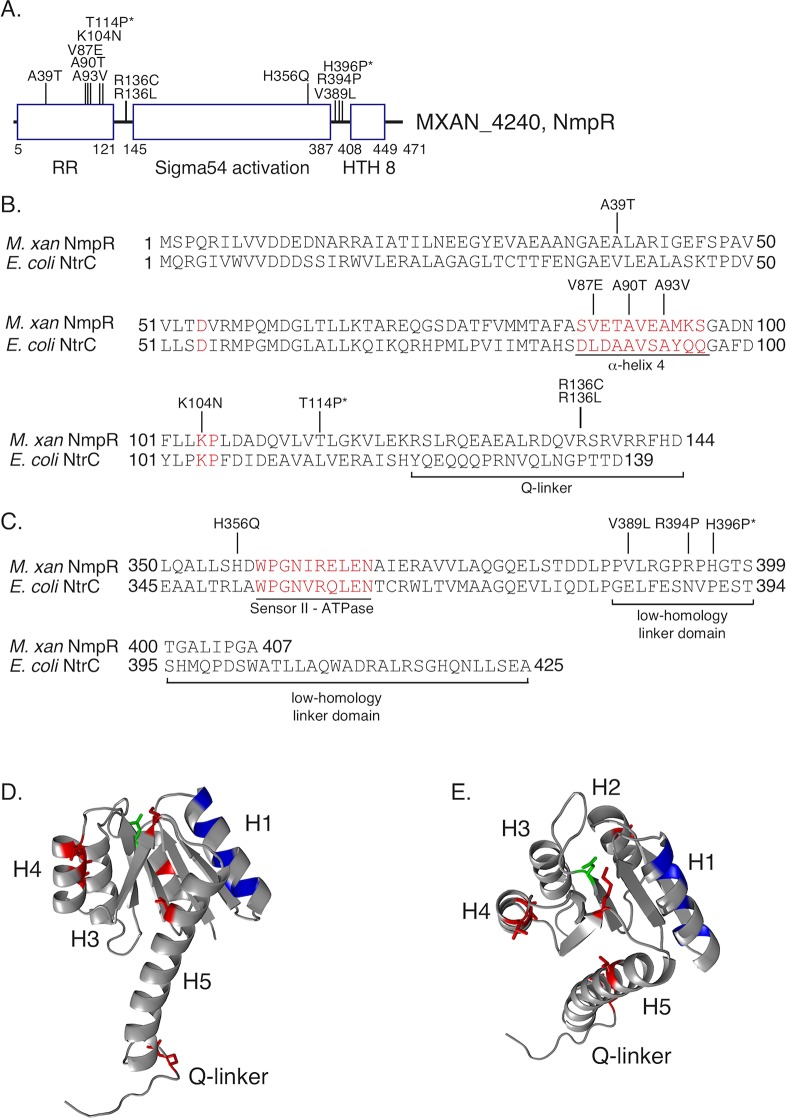
Additional gain-of-function suppressor mutations identified in *nmpR*. The *M*. *xanthus* Δ*pilR* strain was transformed with p_High_-*nmpR*, and suppressor mutants with restored motility were isolated and subjected to targeted sequencing of the over-expression construct. The identified mutations are indicated in a linear depiction of the NmpR domains (A), in the primary amino acid sequence of NmpR (B and C), or unbiasedly modeled on the ribbon structure of NtrX of *Brucella abortus* [51] with α-helices H1-H5 indicated (D and E). In panels B and C, the *E*. *coli* NtrC amino acid sequence is included for comparison and regions of interest in the protein sequence are indicated in red text and/or underlined including: the α-helix 4 of the RR receiver domain, the nearly 100% conserved RR amino acid pair of KP at 104/105, the variable Q-linker, the sensor II motif necessary for ATPase activity, and the low-homology linker between the sigma54 activation domain and helix-turn-helix. Mutations indicated with a * were identified in suppressor mutants resulting from the NmpU Q283Stop Δ*nmpR* strain (See text and Fig 5C). Finally, in panels D and E the same suppressor mutations of NmpR are indicated in red, the conserved aspartic acid that becomes phosphorylated in green, and the specificity residues [11] of NmpR in blue.

During the complementation experiments described above, we were unable to recover transformants expressing the *nmpR* V87E allele expressed from the p_High_ promoter in the absence of an endogenous copy of *nmpR*. This suggested to us that the NmpR gain-of-function is toxic to *M*. *xanthus* when over-produced. Nonetheless, we hypothesized that additional mutations in *nmpR* would lead to a similar gain-of-function and rationalized that over-expression of wild-type *nmpR* in the Δ*pilR* strain would reveal additional suppressor mutations as the endogenous allele of *nmpR* in this strain may provide protection from any detrimental consequences of constitutive activation of the heterologously expressed *nmpR*. As expected, over-expression of *nmpR* in Δ*pilR* did not restore motility, yet after prolonged incubation on 0.5% agar suppressor mutations were readily observed. These suppressor strains were isolated as before and confirmed to have restored T4P-dependent motility on fresh 0.5% agar. Subsequently, we selectively PCR amplified the over-expression *nmpR* construct (with a forward primer in the heterologous promoter to differentiate it from the wild-type allele) and sequenced. From this suppressor screen strategy, we were able to identify an additional nine independent, gain-of-function mutations in *nmpR* (Fig 4). Importantly, in a subset of these strains, we also sequenced the wild-type *nmpR* and *nmpU* loci and found no mutations, indicating the restored motility was due to the identified mutations in the over-expression *nmpR* construct. These mutations clustered in the receiver domain, especially near the phosphorylation pocket, and also in the variable “Q-linker” [45] domain that connects the receiver domain with the central σ^54^ activation domain (Fig 4). Therefore, these mutations likely influence the activation of NmpR by promoting or mimicking the phosphorylated state. Another smaller cluster of mutations was identified near the C-terminus of the σ^54^ activation domain and in the low-homology linker between the activation domain and the helix-turn-helix domain, suggesting that these might change the ATPase activity and/or the DNA binding affinity (Fig 4). Collectively, identification of these suppressor mutations in *nmpR* emphasizes a role for activated NmpR during modulation of *pilA* expression.

### The SK NmpU and RR NmpR are part of a complex signaling pathway with the atypical hybrid SK NmpS

**Fig 5:**
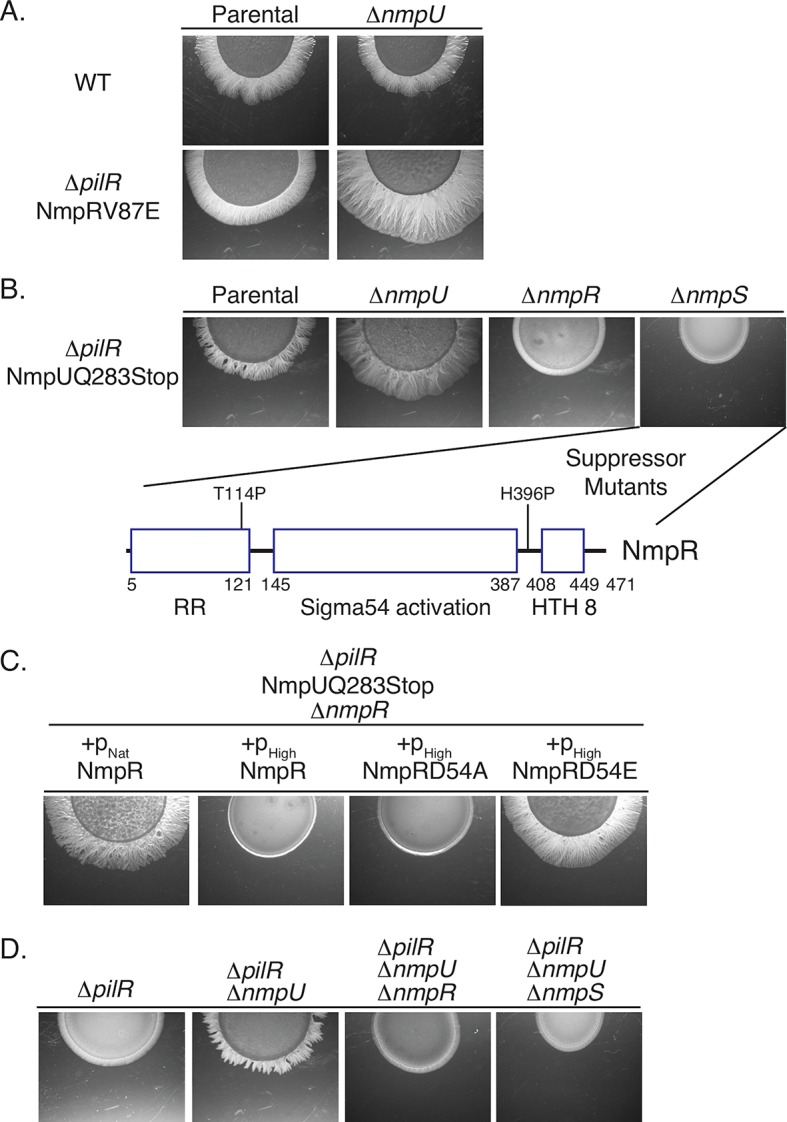
SK NmpU, RR-SK NmpS, and RR NmpR are in a single signaling pathway. Epistasis analysis demonstrates these signaling proteins are in a shared pathway. Mutations in various strains were constructed as depicted and motility assayed as before. Note especially that deletion of *nmpR* or *nmpS* is epistatic to a deleted or non-functional *nmpU* (B and D); that suppressor mutations in *nmpR* arose in the NmpUQ283Stop Δ*nmpS* strain (B and Fig 4); and that deletion of *nmpU* is sufficient to restore motility in the Δ*pilR* parental strain (D). As before, over-expression of a phosphomimetic (D54E) NmpR is necessary to restore motility of a Δ*nmpR* strain (C). The motility image of the parental NmpRV87E strain (A) is a representative image reproduced from Fig 3A.

Nearly all of the initial suppressor mutants identified were in the gene *mxan_4246* encoding a SK that we have designated NmpU. However, within the same genomic locus is another SK that we have designated NmpS. Both of these SK were plausible candidates for cognate kinases of NmpR, so we sought to systematically and genetically determine if these signaling proteins are in a shared pathway. First, we deleted *nmpU* in several genetic backgrounds (Fig 5). Deletion of *nmpU* did not impair motility in wild-type *M*. *xanthus* DZ2 (Fig 5A), consistent with the phenotype of deletion of *nmpR* (Fig 3A). This further emphasizes that in the presence of PilR, neither the SK NmpU or the RR NmpR is necessary for motility and both likely play modulatory roles under certain environmental conditions. In the context of the NmpRV87E suppressor, deletion of *nmpU* did not result in a loss of motility, indicating the V87E alteration of NmpR in this suppressor strain is “blind” to the activity of NmpU (Fig 5A). Finally, deletion of the mutated allele of *nmpU* in the suppressor strain NmpUQ283Stop (Table 1 and Fig 2; C5) did not result in a loss of motility, consistent with the prediction that NmpU is non-functional in this strain (Fig 5B).

To demonstrate NmpU and NmpR are in a shared pathway, we next performed epistasis analysis, combining in-frame *nmpU* and *nmpR* deletions. When *nmpR* was deleted in the NmpUQ283Stop suppressor strain, it caused a return to a non-motile phenotype (Fig 5B). Therefore, Δ*nmpR* is epistatic to the *nmpU* suppressor mutation and is a downstream output of a signaling system containing NmpU. This epistasis was also observed in two additional *nmpU* suppressor strains (NmpUQ61Stop, C2 and NmpUQ217Stop, C4), indicating *nmpR* is the output regardless of the location of the *nmpU* mutation. Next, complementation experiments similar to those presented in Fig 3B were conducted in the NmpUQ283Stop Δ*nmpR* strain (Fig 5C). In this strain, complementation with wild-type *nmpR* expressed from its native promoter was sufficient to restore motility. However, over-expression of the wild-type allele from the p_High_ promoter was not sufficient. We interpreted this to mean that when over-expressed, most of the NmpR would be unphosphorylated, even in the suppressor background. This is consistent with observations mentioned above that the presumed stoichiometry of the active to non-active state of NmpR is critical to influence motility and the wild-type, unphosphorylated NmpR is dominant to the suppressor mutations. Indeed, when an unphosphorylatable *nmpR* (D54A) was over-expressed, motility was not restored, while over-expression of a phosphomimetic *nmpR* (D54E) was sufficient for restored motility (Fig 5C).

Given that there are two additional TCS proteins encoded in the same genomic locus as *nmpU* and *nmpR* (*mxan_4244*, *nmpS*, and *mxan_4245*, *nmpT*; Fig 2A), we sought to test whether the hybrid sensor kinase NmpS also plays a role in a signaling pathway that includes SK NmpU and RR NmpR. Returning to our epistasis analysis, deletion of *nmpS* in the NmpUQ283Stop returned it to a non-motile state (Fig 5B and 5C). Therefore, Δ*nmpS* is also epistatic to the suppressor mutation of *nmpU* and is in a signaling pathway that includes the NmpU and NmpR. Remarkably, in the non-motile NmpUQ283Stop Δ*nmpS* strain (Fig 5B), additional suppressor mutants developed and when sequenced were once again identified in *nmpR*, indicating that NmpR is the output of this multi-component signaling pathway. Activation of NmpR is sufficient to restore motility regardless of the loss of activity of the SKs NmpU or NmpS. Finally, epistasis analysis described here was recapitulated in the parental Δ*pilR* strain; deletion of *nmpU* was sufficient to restore motility, and deletion of *nmpS* or *nmpR* were epistatic to the *nmpU* deletion (Fig 5D).

### Phosphotransfer reveals specificity within the NmpRSTU multi-component system

**Fig 6:**
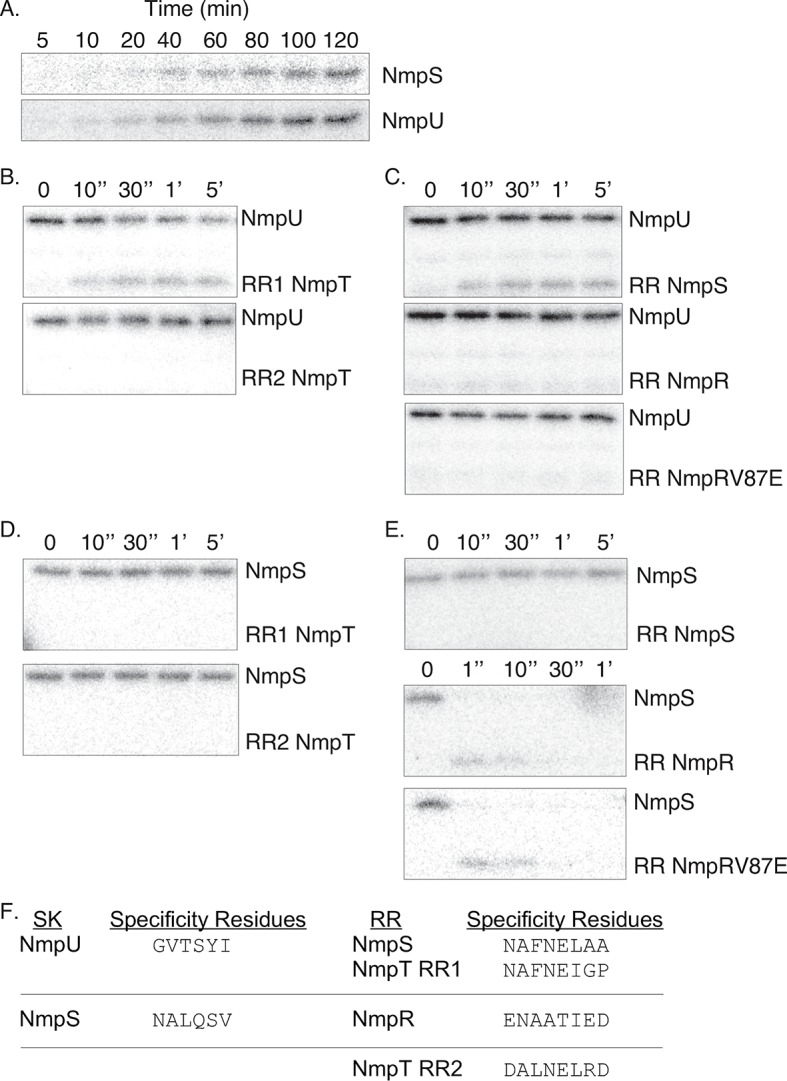
*In vitro* phosphotransfer in the NmpRSTU multi-component pathway. (A) The kinases NmpS and NmpU autophosphorylate in the presence of ATP-γP^32^ as seen by the accumulation of the radioactive phosphoryl group. NmpU specifically phosphorylates the first receiver domain of NmpT (RR1) and the receiver domain of NmpS (B and C). In contrast, NmpS only phosphorylates NmpR (D and E). Note the rapid kinetics of the phosphorylation of NmpR by NmpS (E). Also, the V87E amino acid change of NmpR does not affect the specificity of the phosphotransfer (C and E). The specificity residues [10, 11, 96] of the SK domains of NmpS and NmpU and the corresponding specificity residues of the receiver domains of NmpR, NmpS, and NmpT further supports the observed phosphotransfer patterns (F).

The epistasis analysis described above clearly demonstrated that NmpR, NmpS, and NmpU are in a single signaling pathway. Yet, we could not be sure of the flow of phosphoryl groups and therefore biochemical regulation of the pathway. To conclusively demonstrate which SK is communicating with which RR, we purified each signaling component of the pathway individually as His-tagged protein constructs. To limit the complexity of this initial analysis, only the kinase domains (i.e. no sensor domain) or response regulator receiver domains (i.e. no output domain) were purified. Following well-established protocols based on enzymatic kinase activity and radio-labeled ATP [12], we performed *in vitro* autophosphorylation and phosphotransfer assays (Fig 6). Indeed, both NmpS and NmpU displayed autokinase activity confirming they are active kinases (Fig 6A). These SKs were then individually incubated pair-wise with each RR receiver domain. Based on the epistasis analysis, NmpU is the “top” kinase in the pathway and the phosphotransfer analysis supports this conclusion. NmpU phosphorylated the receiver domain of the hybrid SK NmpS but did not phosphorylate the receiver domain of the final signaling output NmpR (Fig 6C). In addition, NmpU phosphorylated the first receiver domain of the dual receiver domain protein NmpT (Fig 6B), consistent with results presented elsewhere [46] and which we suggest means that NmpT is a phospho-sink of NmpU. In direct contrast to the activity of NmpU, the hybrid SK NmpS only phosphorylated the receiver domain of NmpR (Fig 6D and 6E). NmpS did not phosphorylate its own receiver domain or either of the receiver domains of NmpT. Together the phosphotransfer data suggests signaling fidelity within this branched pathway is dictated by the specificity residues of the SK/RR pairs [11]. The specificity residues of the first receiver domain of NmpT and the receiver domain of NmpS are nearly identical which likely explains the ability of NmpU to phosphorylate both (Fig 6F). On the other hand, the unique specificity residues of NmpR support the observed phosphotransfer by only NmpS. Furthermore, the mutations identified in NmpR are not near the specificity residues (Fig 4D) and the V87E amino acid change of NmpR did not affect the specificity of the pathway. NmpRV87E is still phosphorylated by NmpS, but not by NmpU (Fig 6C and 6D). Thus, our data supports the assertion that the specificity residues determine phosphotransfer specificity even in the context of complex pathways.

### The aspartic acid residue in the receiver domain of NmpS is dispensable for its autokinase activity

**Fig 7:**
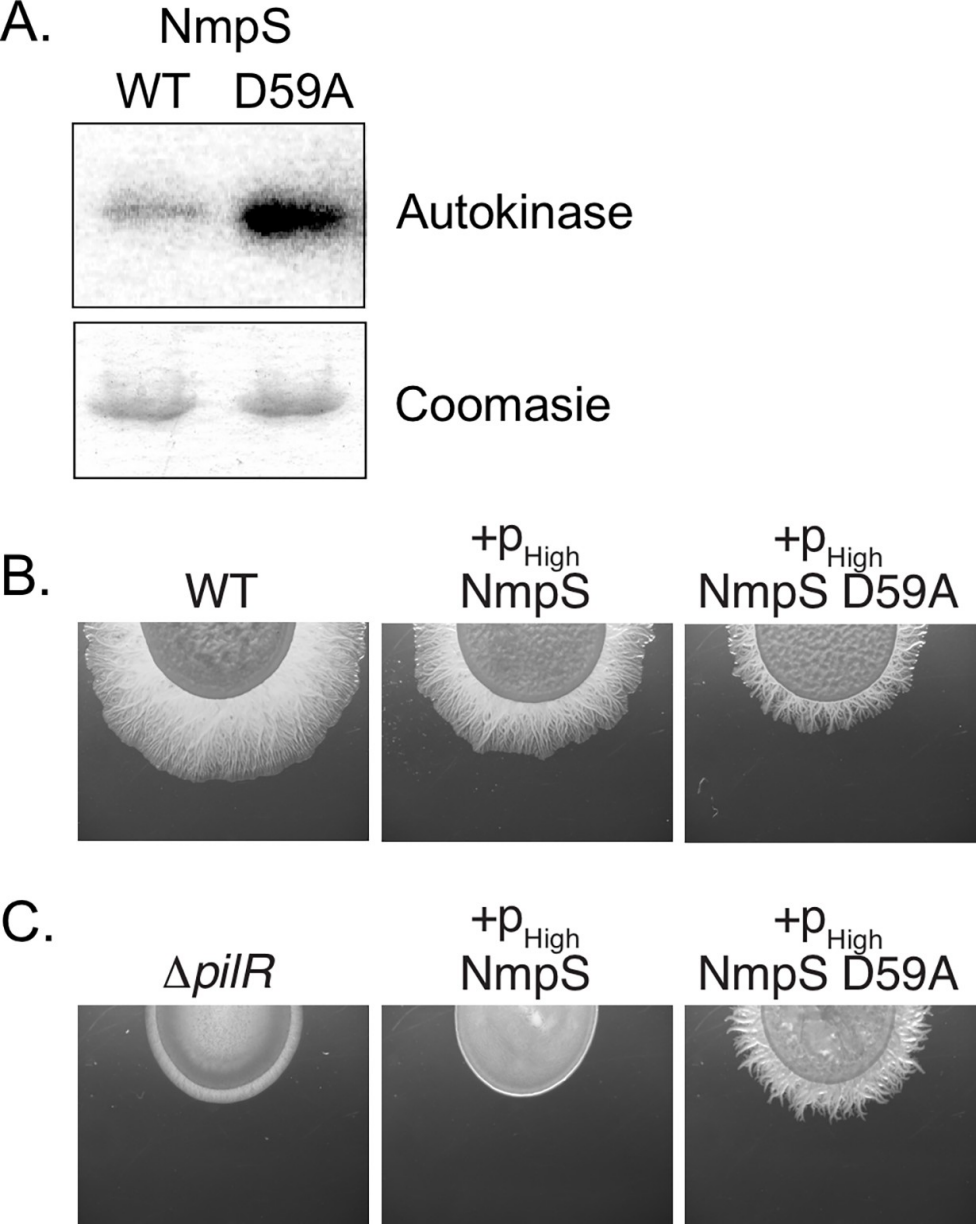
The conserved aspartic acid of the response regulator domain of NmpS is not necessary for kinase activity. (A) Full-length wild-type NmpS and a construct in which the conserved aspartic acid was substituted with an alanine (D59A) were used in an autokinase assay as in Fig 6. The aspartic acid residue was not necessary for activity, supporting the conclusion that the unphosphorylated form of NmpS is the active state. A Coomasie stained gel is included demonstrating the same amount of kinase was in each reaction. (B and C) *M*. *xanthus* wild-type or the Δ*pilR* strain were transformed with NmpS or NmpS D59A over-expression constructs and only the NmpS D59A construct was able to rescue motility of Δ*pilR*. This again is consistent with the *in vitro* kinase activity (A) that supports the conclusion that the unphosphorylated form of NmpS is active.

Based on the epistasis analysis and the phosphotransfer data presented above, we hypothesized that the RR receiver domain of the hybrid kinase NmpS acts as a “sensing domain” and that phosphorylation of this domain by NmpU maintains NmpS in an “off” conformation. To test this, we again took both a biochemical and genetic approach (Fig 7). Full-length NmpS was purified and assayed in autokinase assays as described above. Full-length NmpS does indeed have baseline activity, indicating the amino-terminal RR receiver domain does not prevent autokinase activity *in vitro* (Fig 7A). A NmpS construct containing a substitution at the conserved aspartic acid to alanine displayed greater activity than wild-type. Furthermore, complementation of the Δ*pilR* strain with a NmpS D59A construct was able to restore motility to this strain, while complementation with the full length wild-type NmpS did not (Fig 7C). To date we have been unsuccessful in demonstrating direct regulation of NmpS kinase activity by phosphorylation of its receiver domain *in vitro* and more research will clarify the role of this RR receiver domain in signaling of this atypical SK. Yet, these data are consistent with the conclusion that the aspartic acid and its phosphorylation are not necessary *in vitro* or *in vivo* and that unphosphorylated NmpS (i.e. NmpS D59A) is the more active form of this SK.

### NmpR binds specific promoters upstream of its own putative operon and upstream of *pilR* in the *M*. *xanthus pil* locus

**Fig 8:**
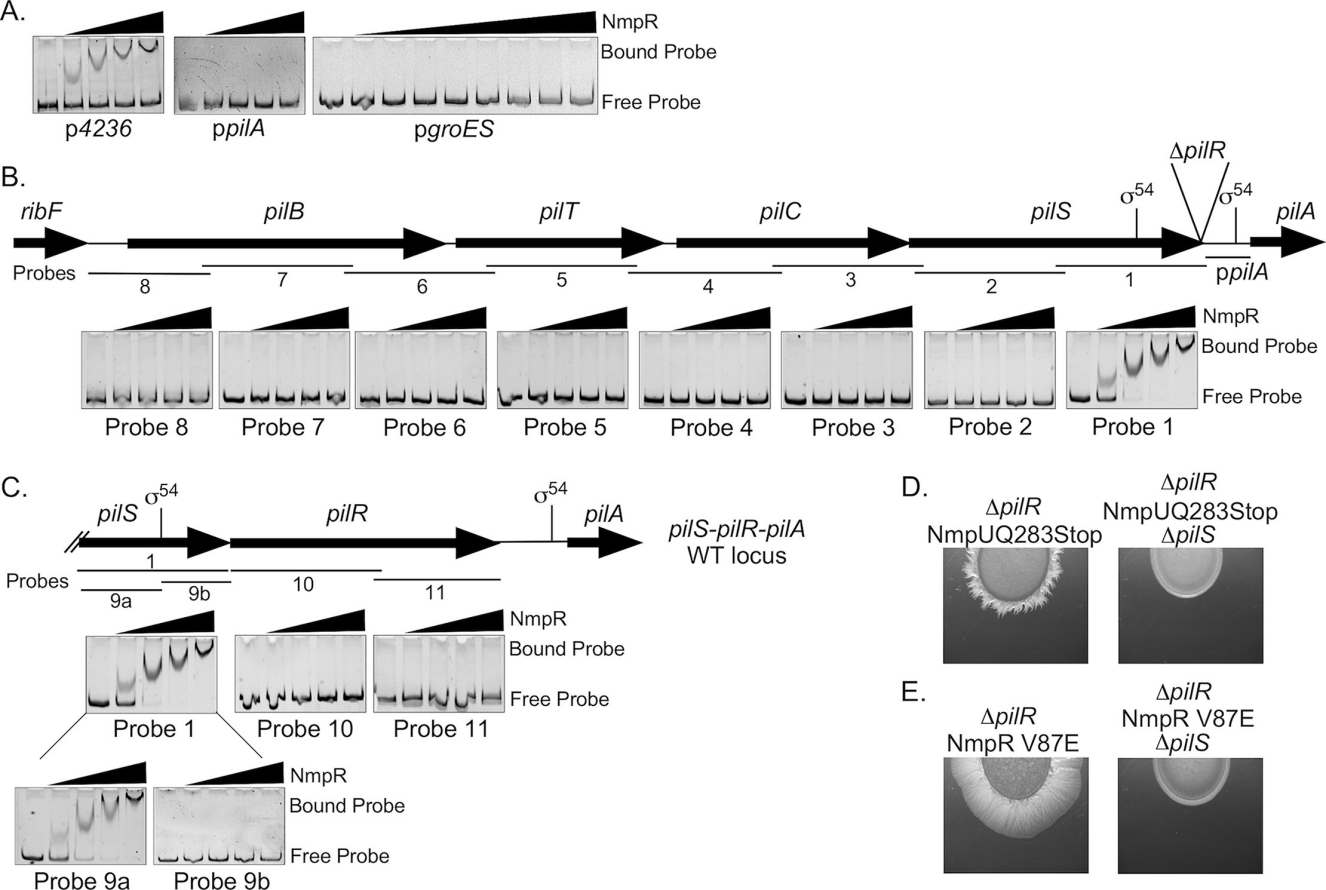
NmpR binds specific promoters upstream of its own putative operon and upstream of *pilR*. (A) NmpR binds to the promoter regions of *mxan_4236* (referred to elsewhere as p_Nat_—585 bp upstream of *mxan_4236*) (See also Fig 2A), but not to that of the *pilA* promoter (217 bp upstream of *pilA*) or p*groES* (referred to elsewhere as pHigh—623 bp upstream of *mxan_4894*, *groES*). (B) NmpR binds specifically to a probe encompassing 800 bp upstream of *pilR* that includes a putative σ^54^ in the *pilS* open reading frame. The σ^54^ in the p*pilA* known to be utilized by PilR in a wild-type genomic context is also indicated. (C) Finally, NmpR binds only to a region within *pilS* upstream of the putative σ^54^ binding site. The sequence containing this NmpR-dependent promoter is necessary for the restored motility in the suppressor strains because deletion of *pilS* returned these strains to a non-motile phenotype (D and E). All shift assays depicted contained 200 fmol of DNA probe and increasing quantities of NmpR (0, 2, 8, 16, and 32 pmol, left to right). The exception is the p*groES* shift that includes the same quantities of NmpR but with a broader range (0, 2, 4, 8, 12, 16, 20, 24, 32 pmol, left to right). All gene lengths and locations, as well as probe length and location are to scale.

Having established the flow of phosphoryl groups through the multicomponent Nmp signaling system with *in vitro* kinase assays and epistasis analysis, we sought to establish the final NmpR output. That is, we tested whether NmpR directly regulates *pilA* or if the resulting suppressor phenotype is facilitated through an alternative indirect mechanism. To answer this question, full-length NmpR was purified and used in electromobility shift assays to determine the DNA regions that NmpR binds (Fig 8). Initially, three probes were designed: the *pilA* promoter (-217 bp upstream of the *pilA* start site), the putative native promoter of *nmpR* upstream of *mxan_4236* (Fig 2A; used for the complementation experiments), and the *groES* promoter as a negative control (again, the same region used for the complementation experiments, p_High_). As expected, increasing concentrations of NmpR did not shift the p*groES* probe. Conversely, a reproducible shift was evident with increasing concentrations of NmpR and the p*4236* probe. This suggests that NmpR is autoregulatory by binding to a promoter upstream of *mxan_4236*. Finally, contrary to our original hypothesis, NmpR did not shift the p*pilA* probe, suggesting that NmpR does not bind this region to directly regulate *pilA* (Fig 8A).

This observation suggested that the regulation of *pilA* in the suppressor strains might be indirect, for example by regulating the expression of a different transcription factor that in turn regulates *pilA*. Alternatively, NmpR could regulate another portion of the *pil* locus that would then explain its ability to restore motility in the suppressor strains, as nearly all genes within the *pil* locus are co-transcribed [26]. Therefore, overlapping 800 bp DNA probes spanning the entire *pil* region upstream of *pilA* were generated and used in electromobility shift assays (Fig 8B). NmpR shifted a probe containing 800 bp immediately upstream of *pilR* was specific; no other probe tested was bound by NmpR. A follow-up assay confirmed that NmpR does not bind within the *pilR* sequence but does bind a sequence upstream of a putative σ^54^ binding site within the *pilS* open reading frame. The potential σ^54^ binding site (TGGCACGTGACGTACG) begins at -351 bp relative to the *pilR* start codon in wild-type *M*. *xanthus* and is positioned -537 bp from the *pilA* start codon in the Δ*pilR* strain. These results suggested that *pilS* contains an internal NmpR-dependent promoter for regulation of *pilR* that is utilized to rescue *pilA* expression and therefore motility in the Δ*pilR* suppressor strains. Indeed, when *pilS* was deleted in two suppressor backgrounds, the strains returned to a non-motile phenotype (Fig 8D and 8E). Therefore the sequence containing the NmpR binding site is necessary for the suppressor phenotypes and strongly suggests that this promoter is NmpR-dependent, highly specific, and physiologically relevant.

### NmpRSTU is a multicomponent signaling pathway that likely functions to regulate *pilR* expression, and therefore *pilA* expression and T4P-dependent motility, in response to oxygen

**Fig 9:**
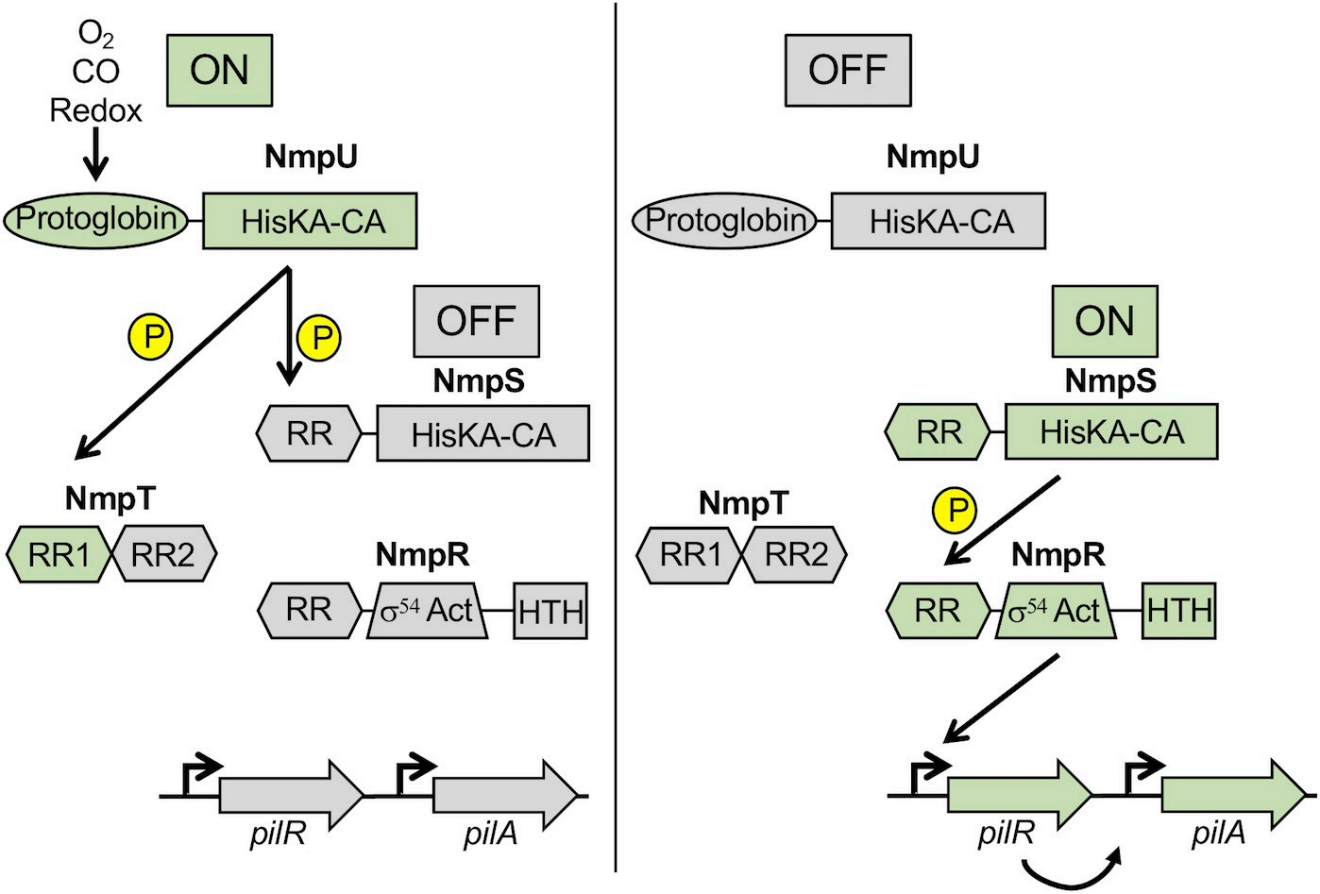
Model of the NmpRSTU multi-component signaling pathway. The presence of oxygen is sensed by the protoglobin domain of the SK NmpU. During high oxygen conditions, NmpU is “on”, autophosphorylating and subsequently phosphorylating the receiver domain of the hybrid RR-SK NmpS. We propose this would maintain NmpS in an “off” state. Additionally, the first receiver domain of NmpT serves as a phospho-sink for NmpU. When oxygen is limiting, perhaps in certain soil types/depths or during multi-cellular development, NmpU switches to an “off” state, leading to the loss of phosphorylation of NmpS and turning “on” the second branch of the pathway. NmpS phosphorylation of the RR NmpR under these conditions activates NmpR leading to modulation of the expression of *pilR*, which in turn regulates *pilA* expression influencing T4P-dependent motility.

In summary, the genetic and biochemical characterization of the NmpRSTU pathway leads us to propose a model (Fig 9) whereby the “top” kinase NmpU phosphorylates NmpS to maintain it in an off state. NmpU also phosphorylates NmpT, though at this time it is unclear the role of NmpT as either a phospho-sink and/or inhibitor of the pathway. In the absence of phosphorylation of the NmpS RR receiver domain, NmpS is active and phosphorylates NmpR, making NmpRS a *bona fide* cognate SK-RR pair. NmpR appears to be autoregulatory, binding to the promoter of *mxan_4236* (Fig 8A) and potentially other promoters throughout the *M*. *xanthus* genome. Critical to the role of NmpR in motility regulation, it binds a specific promoter upstream of *pilR* that we propose increases the relative concentration of PilR that in turn promotes *pilA* expression. Thus, NmpR indirectly modulates *pilA* expression during certain environmental conditions, likely changes in oxygen concentrations sensed by the protoglobin domain of NmpU. Perturbations of this pathway that alters the kinase activity, especially those that promote or mimic the phosphorylated state of NmpR, lead to *pilA* expression that rescues T4P-dependent motility in the absence of the otherwise necessary RR PilR.

## Discussion

The NtrC-like RR family has been extensively studied and has served as a model protein for intramolecular structure-function dynamics [18, 19, 23, 47–50]. NtrC-like RRs also continue to be discovered as important transcriptional regulators controlling diverse biological processes in diverse bacterial species [51–54]. For example, NtrC-like RRs and σ^54^ play critical roles in processes exterior to the bacterial cell, such as lipopolysaccharide production, EPS production, and motility [24]. *M*. *xanthus* encodes at least 27 NtrC-like RR and many of these likely arose by gene duplication during the evolution of ∂-proteobacteria [27]. *M*. *xanthus* NtrC-like RRs are known to regulate such complex behaviors as multicellular fruiting body formation, sporulation, T4P-dependent social motility, and predation [3, 25, 26, 29, 30, 32, 33, 35, 36, 55]. There is also evidence that there are hierarchal and/or overlapping transcriptional programs mediated by these complex TCS networks [3, 33]. Therefore, *M*. *xanthus* is an ideal organism to study how these complex networks regulate the flow of information in a global cellular context.

In this study, we have identified a TCS with no previously described function that includes an NtrC-like RR that we propose indirectly modulates *pilA* expression and therefore T4P-dependent social motility of *M*. *xanthus*. We have named the system NtrC Modulator of Pili (Nmp). Interestingly, the gene encoding NmpR was previously disrupted by insertional mutagenesis and found to have no motility or developmental phenotype [25]. The lack of a phenotype in that background is consistent with our observation that NmpR function was only revealed under standard laboratory conditions via bypass suppressor mutations that arose to restore motility in the absence of PilR. Remarkably, the same selective pressure to restore motility in order to obtain nutrients has been observed elsewhere, such as in *Pseudomonas fluorescens* where a deletion of *fleQ*, an NtrC-like RR necessary for expression of flagellum biosynthesis machinery, was rescued by suppressor mutations in *ntrB* (the SK of the NtrBC system) that lead to hyperphosphorylation of NtrC [56].

The Nmp signaling system described here adds to the already complex picture of gene regulation for the control of social motility in *M*. *xanthus* [26, 34, 35, 57, 58] and is composed of at least four components: the SK NmpU (Mxan_4246), a putative phospho-sink NmpT (Mxan_4245), an atypical hybrid SK NmpS (Mxan_4244), and a final output RR NmpR (Mxan_4240) (Fig 9). Notably, within other members of the ∂-proteobacteria lineage, *nmpRSTU* comprise one contiguous locus (S2 Fig), emphasizing their function as a unit within this clade. In contrast, genomes of more distantly related ∂-proteobacteria (e.g. Desulfovibrionales and Desulfuromonadales orders) contain only a homologue of the SK, NmpU (S2 Fig). Yet, there are examples of homologues of *nmpSTU* in diverse bacterial species including *Planctomyces sp SH-PL14* (S2 Fig). Like *M*. *xanthus*, Planctomycetes are bacteria that have a complex lifecycle, are found in soil, and have extracellular appendages [59, 60]. Collectively, these observations suggest that the NmpRSTU signaling unit has been gained (or lost) throughout evolution within the ∂-proteobacteria and that horizontal gene transfer between species within this clade and between distantly related clades may have been common. As genomic sequences of previously unappreciated bacteria become more common-place and publicly available [61–65], this signaling system may be revealed to be more widespread than currently appreciated.

A homologue of NmpU (AfGcHK) exists within a closely related myxobacterial species, *Anaeromyxobacter sp*. *Fw109-5* (S2 Fig). Autophosphorylation of this kinase occurs when oxygen is bound to an iron molecule found within the heme co-factor of its protoglobin domain (Fig 2C) [66]. *Anaeromyxobacter* species are considered anaerobic, but they likely evolved from an aerobic ancestor within the δ-proteobacteria lineage [67]. Moreover, *Anaeromyxobacter dehalogenans* produced motility flares under aerobic conditions indicating this organism is aerotolerant and may utilize the protoglobin sensor AfGcHK (NmpU) to respond to oxygen concentrations to influence motility. Protoglobin sensors are part of the family of globin-coupled sensors found in the majority of bacterial species and in some archaea and fungi [68–72]. They are known to regulate physiological events in response to oxygen, such as aerotaxis of *Bacillus subtilis* and the archaeon *Halobacterium salinarum* [73]. Thus, we propose that this multi-component signaling system finely tunes the expression of *pilR* (and by extension *pilA*) in response to oxygen concentrations. During oxygen rich conditions, we propose the SK NmpU is “on” and autophosphorylates as the first step of this pathway (Fig 9). It is worth noting that *M*. *xanthus* is thought of as an obligate aerobe and so under laboratory conditions cells are grown and assayed in oxygen replete conditions. We therefore hypothesize NmpU is typically “on” in laboratory settings, keeping NmpR unphosphorylated and unable to affect downstream promoter targets (Fig 9). In natural settings, *M*. *xanthus* must encounter environments with varying oxygen concentrations, perhaps within the soil column or during multicellular development, so therefore likely encodes the ability to respond to an oxygen gradient.

Following autophosphorylation, the NmpU homologue of *A*. *dehalogenans Fw109-5* mentioned above preferentially transfers the phosphoryl group to the first receiver domain of the NmpT homologue [46, 66]. Our analysis fits with that data, as NmpU phosphorylated RR1 of NmpT, but not RR2 (Fig 6B). We expanded the known partners of NmpU by demonstrating that this kinase also phosphorylates the receiver domain of the hybrid SK NmpS which is encoded immediately downstream of NmpT. Remarkably, the specificity residues (amino acids that confer cognate SK/RR specificity [10, 11, 74]) are nearly identical between the RR1 domain of NmpT and the receiver domain of NmpS (Fig 6F) which explains the ability of NmpU to phosphorylate both receiver domains. We propose that phosphorylation of NmpS maintains it in an “off” state (Fig 7). This conclusion is supported in part by the fact that deletion of *nmpS* was epistatic to the null mutations of *nmpU* (Fig 5B and 5D) and that a NmpS D59A construct displayed increased autokinase *in vitro* and was able to rescue motility of the Δ*pilR* strain *in vivo* (Fig 7). Interestingly, hybrid SKs with an amino-terminal receiver domain and carboxyl-terminal histidine kinase domain, such as NmpS (Figs 2 and 7), may utilize the receiver domain as a “sensing domain” that regulates kinase function. For example, the function of a similar hybrid kinase, EsxG of *Rhizobium* NT-26, is controlled via phosphorylation of its amino-terminal receiver domain [75]. Phosphorylation of EsxG is provided by another SK and promotes a closed EsxG conformation that is then unable to phosphorylate its own downstream cognate RR. Additionally, at least one other hybrid kinase with the same domain architecture as NmpS is Lvr, a SK important for virulence of *Leptospira interrogans*. Like NmpS, Lvr retains wild-type levels of autokinase activity when the conserved aspartic acid was substituted with alanine [76]. We expect that the receiver domain of NmpS impacts the conformation of individual monomers of this kinase, but could also affect the oligomeric state of a collection of NmpS monomers. The phosphorylation state and stoichiometry of all components of the Nmp pathway will ultimately determine the SK on/off states of NmpS, and will inform us on the regulation of atypical SKs broadly. These mechanisms of NmpS kinase regulation will be one area of further study.

We propose that the “switch” of the Nmp pathway occurs during oxygen depleted conditions (or when NmpU is mutated to be non-functional) when NmpU no longer phosphorylates NmpS. In the absence of phosphorylation of NmpS, the second branch of the pathway becomes “on”. NmpS is then kinase active, phosphorylates NmpR, leads to modulation of *pilR* and modulates *pilA* expression (Fig 9). The conclusion that NmpR is the output of this multi-component pathway is supported by: 1) deletion of *nmpR* was epistatic to *nmpU* mutations (Fig 5B and 5D), 2) unique *nmpR* suppressor mutations arose in a strain in which NmpU or NmpS was non-functional or deleted, respectively (Fig 5C), 3) attempts to isolate further suppressor mutations in a Δ*pilR*Δ*nmpR* strain were unsuccessful, and 4) NmpR specifically binds a DNA region upstream of a σ^54^ binding site in proximity to the *pilR* start codon that is necessary for the suppressor phenotype (Fig 8).

In further support of our model, we demonstrate that NmpSR must be “on” to modulate *pilR* expression (Fig 9). First, suppressor mutations that seem to confer activation of the RR NmpR allowed for restored motility (Fig 4). One of these activating mutations that we characterized in depth, V87E, was necessary and sufficient for the restored motility (Fig 3). Furthermore, the NmpR D54A allele that cannot be phosphorylated is unable to restore motility while the phosphomimetic NmpR D54E is sufficient to restore motility (Fig 3B and Fig 5D). Second, suppressor mutations that confer activation of NmpR to restore motility (including V87E) were identified in the well-conserved α-helix 4 within the RR receiver domain which contains several surface exposed amino acids (Fig 4). Structural and functional analysis of both NtrC and RRs in general, have indicated that this helix undergoes significant conformational changes between the inactive and active state, making close intermolecular contacts with the SK [77] and direct intramolecular contacts with the σ^54^ activation ATPase domain and dimer interface [18, 19, 47–50, 78]. Indeed, NmpR V87E was phosphorylated by NmpS *in vitro*, and is sufficient to restore motility *in vivo*. Collectively, the data suggests that the V87E mutation promotes the activated state of NmpR.

Another key mutation suggests that the constitutively active form of NmpR promotes motility. Specifically, the K104N mutation (Fig 4) occurs at a position that is nearly 100% conserved in all RR, regardless of family. This lysine forms hydrogen bonds with the phosphoryl group and is critical for the ability to turn the RR “off” (i.e. be dephosphorylated). Alteration at this lysine position to a related but distinct amino acid, such as asparagine or arginine, allows the RR to be phosphorylated but not dephosphorylated. For example, when this conserved lysine of *E*. *coli* CheY (K105) is altered to an arginine (K105R) it has significantly reduced dephosphorylation rates and remains in the “on” state [79, 80]. Similar mutational analysis at the same lysine position has also been demonstrated in NtrC [81, 82].

Importantly, we observed several additional mutations in NmpR that activate motility but have not been identified or characterized for activation of RRs in other organisms. Future studies of how these mutations confer activation to this NtrC-like RR will be important in our understanding of bacterial gene regulation, generally, but also in the context of important RR virulence factors and our potential in targeting these proteins with novel antibiotics [83, 84]. Finally, given several notable properties of this newly described pathway, future investigations have the potential to further our understanding of the dynamics of complex TCS signaling, atypical regulation of hybrid SKs, functions of protoglobin containing sensors, and global cellular NtrC-like RR networks.

## Materials and methods

### Bacterial strains and growth

All *M*. *xanthus* strains are listed in Table S1. *E*. *coli* DH5α and TOP10 (Life Technologies, Grand Island, NY) strains were used for cloning and grown in LB (Becton-Dickinson, Franklin Lakes, NJ) at 37°C. *E*. *coli* BL21 DE3 (Life Technologies, Grand Island, NY) was used for protein production and were grown in Terrific Broth. *M*. *xanthus* strains were cultivated in Casitone Yeast Extract (CYE) medium [85] at 32°C with shaking at 220 rpm. *M*. *xanthus* was grown on agar plates at 1.5% agar, except in motility assays where 0.5% agar was used. For all experiments, the *M*. *xanthus* DZ2 strain was used as the wild-type strain [86]. Kanamycin was used at a final concentration of 50 μg ml^-1^, tetracycline at 10 μg ml^-1^, and spectinomycin at 100 μg ml^-1^ (*E*. *coli*) or 1 mg ml^-1^ (*M*. *xanthus*), when required.

### DNA handling and sequence analysis

PCR reactions were performed using Failsafe polymerase with Buffer K (Epicentre Technologies, Madison, WI) or high-fidelity Phusion polymerase with the manufactured suggested protocol (New England Biolabs, Ipswich, MA). All primers (Table S2) were supplied by Integrated DNA Technologies (Coralville, IA) and were designed based on sequences obtained from the Microbial Signal Transduction Database (MIST2.2, [87]). PCR products used for cloning or deletion mutant construction were initially cloned into the pCR2.1-TOPO vector (Life Technologies, Grand Island, NY) and sequence verified (Nevada Genomics Sequencing Center, Reno, Nevada). DNAStar software (Lasergene 7.2) was used for sequence analysis. Whole genome sequencing of *M*. *xanthus* DZ2, the Δ*pilR* parental strain, and all suppressor mutants reported in this paper was performed by the University of Iowa Institute of Human Genetics, Genomics Division on the Illumina MiSeq Sequencer with 2x250 bp reads. DNAStar software (Lasergene 7.2) was used for genome alignment and mutation identification based on the *M*. *xanthus* DK1622 annotated genome [27]. The mean genomic coverage for all samples was >60x and suppressor mutations identified by a 95% cut-off. Identified mutations were confirmed by traditional Sanger-sequencing methods.

### Construction and complementation of mutants

In-frame deletions of *M*. *xanthus* were constructed essentially as described elsewhere [26, 88]. Briefly, to construct in-frame deletions, approximately 800 bp fragments upstream and downstream of each gene to be deleted was amplified and cloned into pBJ113 [89]. Final plasmid constructs were electroporated into appropriate strains of *M*. *xanthus* [90]. Transformants were selected on CYE agar with kanamycin and screened for proper insertion of the plasmid by PCR, followed by counterselection on galactose [91]. All final deletion mutants were verified by PCR using primers spanning the deletion junction and/or internal to the gene deletion. Complementation of each gene was achieved by cloning the entire opening reading frame down-stream of the heterologous high-expression *groES* promoter (623 bp upstream of *mxan_4894* [43, 44]; S1 Fig) or, the putative native promoter of *nmpR* (585 bp upstream of *mxan_4236*; Fig 2A). Mutagenesis of vector constructs was done with QuikChange Site-Directed Mutagenesis Kit (Agilent Technologies) or Q5 Site-Directed Mutagenesis Kit (New England Biolabs) per the manufacturer’s instructions and constructed mutations sequenced verified. The plasmid pSUM117 was used to integrate the complementation constructs into the Mx9 phage attachment site. This plasmid is a pBCKS+ derivative that contains the attPMx9 site for integration. Final clones were again verified by PCR.

### T4P-dependent motility and motility-related phenotypic analysis

T4P-dependent motility assays were performed as previously described [40]. Briefly, cultures were grown overnight to log phase. Cells were washed in MMC buffer (20 mM MOPS, pH 7.6, 4.0 mM MgSO_4_, 2 mM CaCl_2_), suspended in MMC to ~2x10^9^ CFU ml^-1^, and 10 μl cell suspensions spotted onto dry CYE 0.5% agar plates for growth/motility at 32°C. Overall motility rates were determined by measuring the diameter of the spreading colony at 24 hour intervals for five days. The reported rates reflect the average slope of at least three colonies. An unpaired t-test was used to analyze the significance of the differences of motility rate relative to wild-type or Δ*pilR* motility (p<0.05 considered significant). Motility assays were monitored by microscopy using a Nikon SMZ10000 dissecting microscope. Images were taken using a QImaging Micropublisher charge-coupled device camera and processed with QCapture software. While we attempted to replicate motility conditions between samples, the experiments presented in this study were not all done at the same time. Individual images are representative of multiple observations. To assess the sedimentation of *M*. *xanthus* cells, assays were conducted as described previously [35] by measuring the optical density at 600 nm of standing cell suspensions prepared as for the motility assays. The reported sedimentation percentage is the remaining optical density relative to the original optical density of each strain after two hours. Quantitative measurement of EPS production was conducted as previously described [92] by again preparing cell suspensions in MMC buffer to ~2x10^9^ CFU ml^-1^ with a final concentration of 30 μg ml^-1^ trypan blue in each preparation. The trypan blue was incubated with the cells for 30 min at room temperature, at which time the cells were pelleted and the supernatant removed. Remaining trypan blue in the supernatant was measured by absorbance at 585 nm. Initial trypan absorbance was measured with control samples of trypan blue in which no cells were included. Relative amount of trypan blue binding for each strain was compared to wild-type, which was set at 100%.

### Detection of PilA by western blot

*M*. *xanthus* strains were grown overnight to log phase, pelleted, suspended in 10 mM Tris-HCl pH 7.5, and lysed by sonication. Total protein content was determined by Bradford assay and cell lysates were loaded on a 12% SDS-PAGE gel normalized to total protein concentration. After electrophoresis, the gel was blotted onto a PVDF membrane, blocked with 5% skim milk, and incubated with anti-PilA antiserum [35] at 1:5000 followed by secondary anti-rabbit horseradish-peroxidase-coupled antibody (Sigma) at 1:10,000. Detection was performed using Western Lightning-ECL (PerkinElmer).

### β-galactosidase assay

To assess the activity of the *groES* promoter, the same promoter used for complementation experiments was incorporated upstream of *lacZ*. This plasmid is a pSUM117 derivative containing a *lacZ* reporter and attPMx9 attachment site for integration. Incorporation of the plasmid in single copy at the Mx9 phage attachment site was confirmed by PCR. Expression of *groES* in the resulting strains was assessed following lawn growth on CYE plates for four hours. Cells were scraped from the multiple plates and pooled. To quantify β-galactosidase activity, bacteria were pelleted, suspended in Z-buffer, and lyzed by sonication. Total protein concentration was determined with Bradford reagent and spectrophotometry. Activity was quantified by incubation of normalized cell lysates at 37°C with o-nitrophenol and presented as Miller units ((OD_420_/mg protein * time in minutes)*1000).

### Protein purification and in vitro phosphotransfer experiments

All proteins were purified using a standard column purification protocol [12]. All proteins were expressed using the isopropyl-β-D-thiogalactopyranoside (IPTG)-inducible vector pET28a (Novagen). For purification, *E*. *coli* BL21 DE3 strains containing an expression vector was grown in 100 ml Terrific Broth in a 250-ml Erlenmeyer flask at 37°C with protein expression induced with 1 mM IPTG. After overnight incubation at 16°C, cells were pelleted at 8,000g for 15 min. When necessary, pellets were stored at -20°C until purification. Cell pellets were thawed, suspended in 10 ml of cell lysis buffer (25 mM Tris pH 7.6, 125 mM NaCl, 5 mM imidazole, 1% Triton X-100, and 0.625 g of CelLytic Express (Sigma-Aldrich). Cells were incubated for 1 h before lysates were clarified by centrifugation at 8,000 g. One ml of His-Select cobalt affinity resin (Sigma-Aldrich) was equilibrated in wash buffer (25 mM Tris pH 7.6, 125 mM NaCl, 5 mM imidazole, 1% Triton X-100). Whole cell lysates were equilibrated over the resin by gravity, washed once with 10 ml of wash buffer, twice with 10 ml wash buffer with 20 mM imidazole, and finally eluted with 5 ml of elution buffer (25 mM Tris [pH 7.6], 125 mM NaCl, 250 mM imidazole). Samples were dialyzed overnight at 4°C against dialysis buffer (25 mM Tris pH 7.6, 125 mM NaCl, 1 mM dithiothreitol, 1% Triton X-100, 50% glycerol, 0.5 mM EDTA). Protein purity was assessed by standard denaturing gel electrophoresis, and concentration determined using the Bradford reagent. All purified proteins were stored in dialysis buffer at -20°C.

Kinase assays were performed as described previously [12]. Briefly, 5 μl of 50 μM SK stock was added to 5 μl 10x kinase buffer (250 mM Tris [pH 7.6], 500 mM KCl, 10 mM MgCl2, 10 mM MnCl2, 10 mM CaCl2 and 10 mM β-mercaptoethanol) and 35 μl dH2O. Reaction mixtures were incubated at room temperature and started by the addition of 5 μl of an ATP mix (250 μM ATP, 3 μM [γ-^32^P]ATP). Aliquots were removed at various time points and stopped by the addition of an equal volume of 4x SDS loading buffer. Samples were resolved by electrophoresis on 12% SDS-polyacrylamide gels. Gels were exposed for 3 hours to a phosphor screen and visualized using a Typhoon Imager (GE). For phosphotransfer profiling, kinases were allowed to autophosphorylate for two hours until maximal phosphorylation levels were reached. A 5 μl aliquot of the phosphorylated SK was mixed with 5 μl of 10 μM RR, and reactions were stopped at various times by addition of 4x SDS loading buffer. Incorporation of labeled phosphoryl groups was analyzed as detailed above and figure images are representative of multiple experiments.

### Electromobility shift assays

Full-length wild-type NmpR was expressed, purified, and quantified as above. DNA probes were generated by PCR with Failsafe polymerase with Buffer K (Epicentre Technologies, Madison, WI), purified with Qiagen QIAquick PCR Purification Kit, and quantified by spectrophotometry. Probes were diluted in 10 mM Tris-HCl, pH8.5 to 100 fmol/μl with a final quantity of 200 fmol used per individual binding reaction. Probes were incubated with increasing concentrations of NmpR, as indicated, in a reaction containing a final concentration of 20 mM Hepes pH 7.6, 1 mM EDTA, 10 mM (NH_4_)_2_SO_4_, 1 mM DTT, Tween 20 0.2%, and 30 mM KCl. Reaction conditions were determined empirically in our laboratory but were based on recommendations from the DIG Gel Shift Kit (Sigma-Aldrich). NmpR was incubated with the DNA probes for 15 minutes at room temperature and then loaded on 8% acrylamide non-denaturing PAGE gels (8% acrylamide, 10 mM Tris-HCl, 1 mM EDTA, 400 mM glycine). Gels were resolved at 60 v for ~2 hours, then stained with ethidium bromide (10 μg per 10 ml H_2_O) for 15 minutes prior to visualization. Presented images are representative of reproducible independent reactions.

### Bioinformatic identification of TCS homologues

Several online software platforms were utilized to identify homologues of NmpRSTU. Protein sequences and some synteny analysis were obtained from the Mist 2.2 database [87]. Homologues were also identified by blastp (blast.ncbi.nlm.nih.gov) against δ-proteobacteria (taxid:28221) specifically, or by excluding δ-proteobacteria. Homologues and synteny were also identified at Pfam (pfam.xfam.org; [93]) and SyntTax (archaea.u-psud.fr/synttax/; [94]). The structure of NmpR was unbiasedly modeled using SWISS-MODEL (swissmodel.expasy.org) [95].

## Supporting information

S1 Table Strains and plasmids used in this study. (DOCX)

S2 Table Primers used in this study. (DOCX)

S1 Fig Expression from the *groES* promoter. To determine if the *groES* promoter was reasonable to use for complementation experiments, a *groES*-*lacZ* reporter was constructed and transformed into relevant strains used in this research. The Miller Units reported in this Figure are directly comparable to the levels of the *pilA* reporter: ((OD_420_/mg protein * time in minutes)*1000). The levels of *groES* expression are essentially identical between the strains tested here, when assayed following growth on a hard agar surface.
(TIFF)

S2 Fig Homologues of NmpR (Mxan_4240), NmpS (Mxan_4244), NmpT (Mxan_4245), and NmpU (Mxan_4246) exist in other species with shared synteny. Some members of the ∂-proteobacteria contain homologues of the multi-component signaling pathway (NmpR = blue, NmpSTU in shades of red) (A). Mxan_4236 (purple) is a CBS domain protein and serves as a convenient landmark of the genomic location (A). Only a NmpU homologue could be identified in more distantly related ∂-proteobacteria (B). Shared synteny was also identified outside of the ∂-proteobacteria (C) and multiple examples of NmpU/NmpT homologues (D) were identified outside of the ∂-proteobacteria. Those species identified with a * are all members of the Planctomycetes phylum. Collectively, these observations suggest these genes have been exchanged by horizontal gene transfer followed by divergent evolution of domain architectures.
(TIFF)

## References

[1] Zschiedrich CP, Keidel V, Szurmant H. Molecular Mechanisms of Two-Component Signal Transduction. J Mol Biol. 2016;428(19):3752–75. 10.1016/j.jmb.2016.08.003 ; PubMed Central PMCID: PMCPMC5023499. 27519796 PMC5023499

[2] Bretl DJ, Demetriadou C, Zahrt TC. Adaptation to environmental stimuli within the host: two-component signal transduction systems of Mycobacterium tuberculosis. Microbiol Mol Biol Rev. 2011;75(4):566–82. 10.1128/MMBR.05004-11 ; PubMed Central PMCID: PMCPMC3232741. 22126994 PMC3232741

[3] Sarwar Z, Garza AG. Two-Component Signal Transduction Systems That Regulate the Temporal and Spatial Expression of Myxococcus xanthus Sporulation Genes. J Bacteriol. 2015;198(3):377–85. 10.1128/JB.00474-15 ; PubMed Central PMCID: PMCPMC4719452. 26369581 PMC4719452

[4] Vogt SL, Raivio TL. Just scratching the surface: an expanding view of the Cpx envelope stress response. FEMS Microbiol Lett. 2012;326(1):2–11. 10.1111/j.1574-6968.2011.02406.x . 22092948

[5] Hobbs M, Collie ES, Free PD, Livingston SP, Mattick JS. PilS and PilR, a two-component transcriptional regulatory system controlling expression of type 4 fimbriae in Pseudomonas aeruginosa. Mol Microbiol. 1993;7(5):669–82. . 8097014 10.1111/j.1365-2958.1993.tb01158.x

[6] Ishimoto KS, Lory S. Identification of pilR, which encodes a transcriptional activator of the Pseudomonas aeruginosa pilin gene. J Bacteriol. 1992;174(11):3514–21. ; PubMed Central PMCID: PMCPMC206036. 1317379 10.1128/jb.174.11.3514-3521.1992 PMC206036

[7] Egger LA, Inouye M. Purification and characterization of the periplasmic domain of EnvZ osmosensor in Escherichia coli. Biochem Biophys Res Commun. 1997;231(1):68–72. 10.1006/bbrc.1996.6007 . 9070221

[8] Kato A, Groisman EA, Howard Hughes Medical I. The PhoQ/PhoP regulatory network of Salmonella enterica. Adv Exp Med Biol. 2008;631:7–21. . 18792679 10.1007/978-0-387-78885-2_2

[9] Krell T, Lacal J, Busch A, Silva-Jimenez H, Guazzaroni ME, Ramos JL. Bacterial sensor kinases: diversity in the recognition of environmental signals. Annu Rev Microbiol. 2010;64:539–59. 10.1146/annurev.micro.112408.134054 . 20825354

[10] Skerker JM, Perchuk BS, Siryaporn A, Lubin EA, Ashenberg O, Goulian M, et al Rewiring the specificity of two-component signal transduction systems. Cell. 2008;133(6):1043–54. 10.1016/j.cell.2008.04.040 ; PubMed Central PMCID: PMCPMC2453690. 18555780 PMC2453690

[11] Capra EJ, Laub MT. Evolution of two-component signal transduction systems. Annu Rev Microbiol. 2012;66:325–47. 10.1146/annurev-micro-092611-150039 ; PubMed Central PMCID: PMCPMC4097194. 22746333 PMC4097194

[12] Willett JW, Tiwari N, Muller S, Hummels KR, Houtman JC, Fuentes EJ, et al Specificity residues determine binding affinity for two-component signal transduction systems. MBio. 2013;4(6):e00420–13. 10.1128/mBio.00420-13 ; PubMed Central PMCID: PMCPMC3892784. 24194534 PMC3892784

[13] Kustu S, Santero E, Keener J, Popham D, Weiss D. Expression of sigma 54 (ntrA)-dependent genes is probably united by a common mechanism. Microbiol Rev. 1989;53(3):367–76. ; PubMed Central PMCID: PMCPMC372741. 2677638 10.1128/mr.53.3.367-376.1989 PMC372741

[14] Ninfa AJ, Magasanik B. Covalent modification of the glnG product, NRI, by the glnL product, NRII, regulates the transcription of the glnALG operon in Escherichia coli. Proc Natl Acad Sci U S A. 1986;83(16):5909–13. ; PubMed Central PMCID: PMCPMC386406. 2874557 10.1073/pnas.83.16.5909 PMC386406

[15] Bush M, Dixon R. The role of bacterial enhancer binding proteins as specialized activators of sigma54-dependent transcription. Microbiol Mol Biol Rev. 2012;76(3):497–529. 10.1128/MMBR.00006-12 ; PubMed Central PMCID: PMCPMC3429621. 22933558 PMC3429621

[16] Contreras A, Drummond M. The effect on the function of the transcriptional activator NtrC from Klebsiella pneumoniae of mutations in the DNA-recognition helix. Nucleic Acids Res. 1988;16(9):4025–39. ; PubMed Central PMCID: PMCPMC336572. 3287338 10.1093/nar/16.9.4025 PMC336572

[17] Morett E, Segovia L. The sigma 54 bacterial enhancer-binding protein family: mechanism of action and phylogenetic relationship of their functional domains. J Bacteriol. 1993;175(19):6067–74. ; PubMed Central PMCID: PMCPMC206698. 8407777 10.1128/jb.175.19.6067-6074.1993 PMC206698

[18] Batchelor JD, Doucleff M, Lee CJ, Matsubara K, De Carlo S, Heideker J, et al Structure and regulatory mechanism of Aquifex aeolicus NtrC4: variability and evolution in bacterial transcriptional regulation. J Mol Biol. 2008;384(5):1058–75. 10.1016/j.jmb.2008.10.024 . 18955063

[19] De Carlo S, Chen B, Hoover TR, Kondrashkina E, Nogales E, Nixon BT. The structural basis for regulated assembly and function of the transcriptional activator NtrC. Genes Dev. 2006;20(11):1485–95. 10.1101/gad.1418306 ; PubMed Central PMCID: PMCPMC1475761. 16751184 PMC1475761

[20] Lee SY, De La Torre A, Yan D, Kustu S, Nixon BT, Wemmer DE. Regulation of the transcriptional activator NtrC1: structural studies of the regulatory and AAA+ ATPase domains. Genes Dev. 2003;17(20):2552–63. 10.1101/gad.1125603 ; PubMed Central PMCID: PMCPMC218149. 14561776 PMC218149

[21] Weiss DS, Batut J, Klose KE, Keener J, Kustu S. The phosphorylated form of the enhancer-binding protein NTRC has an ATPase activity that is essential for activation of transcription. Cell. 1991;67(1):155–67. . 1833069 10.1016/0092-8674(91)90579-n

[22] Popham DL, Szeto D, Keener J, Kustu S. Function of a bacterial activator protein that binds to transcriptional enhancers. Science. 1989;243(4891):629–35. . 2563595 10.1126/science.2563595

[23] Rappas M, Bose D, Zhang X. Bacterial enhancer-binding proteins: unlocking sigma54-dependent gene transcription. Curr Opin Struct Biol. 2007;17(1):110–6. 10.1016/j.sbi.2006.11.002 . 17157497

[24] Francke C, Groot Kormelink T, Hagemeijer Y, Overmars L, Sluijter V, Moezelaar R, et al Comparative analyses imply that the enigmatic Sigma factor 54 is a central controller of the bacterial exterior. BMC Genomics. 2011;12:385 10.1186/1471-2164-12-385 ; PubMed Central PMCID: PMCPMC3162934. 21806785 PMC3162934

[25] Caberoy NB, Welch RD, Jakobsen JS, Slater SC, Garza AG. Global mutational analysis of NtrC-like activators in Myxococcus xanthus: identifying activator mutants defective for motility and fruiting body development. J Bacteriol. 2003;185(20):6083–94. 10.1128/JB.185.20.6083-6094.2003 ; PubMed Central PMCID: PMCPMC225022. 14526020 PMC225022

[26] Bretl DJ, Muller S, Ladd KM, Atkinson SN, Kirby JR. Type IV-pili dependent motility is co-regulated by PilSR and PilS2R2 two-component systems via distinct pathways in Myxococcus xanthus. Mol Microbiol. 2016;102(1):37–53. 10.1111/mmi.13445 . 27393239

[27] Goldman BS, Nierman WC, Kaiser D, Slater SC, Durkin AS, Eisen JA, et al Evolution of sensory complexity recorded in a myxobacterial genome. Proc Natl Acad Sci U S A. 2006;103(41):15200–5. 10.1073/pnas.0607335103 ; PubMed Central PMCID: PMCPMC1622800. 17015832 PMC1622800

[28] Giglio KM, Zhu C, Klunder C, Kummer S, Garza AG. The enhancer binding protein Nla6 regulates developmental genes that are important for Myxococcus xanthus sporulation. J Bacteriol. 2015;197(7):1276–87. 10.1128/JB.02408-14 ; PubMed Central PMCID: PMCPMC4352671. 25645554 PMC4352671

[29] Diodati ME, Ossa F, Caberoy NB, Jose IR, Hiraiwa W, Igo MM, et al Nla18, a key regulatory protein required for normal growth and development of Myxococcus xanthus. J Bacteriol. 2006;188(5):1733–43. 10.1128/JB.188.5.1733-1743.2006 ; PubMed Central PMCID: PMCPMC1426557. 16484184 PMC1426557

[30] Giglio KM, Caberoy N, Suen G, Kaiser D, Garza AG. A cascade of coregulating enhancer binding proteins initiates and propagates a multicellular developmental program. Proc Natl Acad Sci U S A. 2011;108(32):E431–9. 10.1073/pnas.1105876108 ; PubMed Central PMCID: PMCPMC3156219. 21670274 PMC3156219

[31] Ossa F, Diodati ME, Caberoy NB, Giglio KM, Edmonds M, Singer M, et al The Myxococcus xanthus Nla4 protein is important for expression of stringent response-associated genes, ppGpp accumulation, and fruiting body development. J Bacteriol. 2007;189(23):8474–83. 10.1128/JB.00894-07 ; PubMed Central PMCID: PMCPMC2168950. 17905995 PMC2168950

[32] Sarwar Z, Garza AG. The Nla28S/Nla28 two-component signal transduction system regulates sporulation in Myxococcus xanthus. J Bacteriol. 2012;194(17):4698–708. 10.1128/JB.00225-12 ; PubMed Central PMCID: PMCPMC3415486. 22753068 PMC3415486

[33] Bretl DJ, Kirby JR. Molecular Mechanisms of Signaling in Myxococcus xanthus Development. J Mol Biol. 2016;428(19):3805–30. 10.1016/j.jmb.2016.07.008 . 27430596

[34] Lancero H, Caberoy NB, Castaneda S, Li Y, Lu A, Dutton D, et al Characterization of a Myxococcus xanthus mutant that is defective for adventurous motility and social motility. Microbiology. 2004;150(Pt 12):4085–93. 10.1099/mic.0.27381-0 . 15583161

[35] Wu SS, Kaiser D. Regulation of expression of the pilA gene in Myxococcus xanthus. J Bacteriol. 1997;179(24):7748–58. ; PubMed Central PMCID: PMCPMC179738. 9401034 10.1128/jb.179.24.7748-7758.1997 PMC179738

[36] Willett JW, Kirby JR. CrdS and CrdA comprise a two-component system that is cooperatively regulated by the Che3 chemosensory system in Myxococcus xanthus. MBio. 2011;2(4). 10.1128/mBio.00110-11 ; PubMed Central PMCID: PMCPMC3147164. 21810965 PMC3147164

[37] Wu SS, Kaiser D. Genetic and functional evidence that Type IV pili are required for social gliding motility in Myxococcus xanthus. Mol Microbiol. 1995;18(3):547–58. . 8748037 10.1111/j.1365-2958.1995.mmi_18030547.x

[38] Jin S, Ishimoto KS, Lory S. PilR, a transcriptional regulator of piliation in Pseudomonas aeruginosa, binds to a cis-acting sequence upstream of the pilin gene promoter. Mol Microbiol. 1994;14(5):1049–57. . 7715443 10.1111/j.1365-2958.1994.tb01338.x

[39] Hodgkin J, Kaiser D. Genetics of Gliding Motility in Myxococcus xanthus (Myxobacterales): Two Gene Systems Control Movement. Molec gen Genet. 1979;171:177–91.

[40] Shi W, Zusman DR. The two motility systems of Myxococcus xanthus show different selective advantages on various surfaces. Proc Natl Acad Sci U S A. 1993;90(8):3378–82. ; PubMed Central PMCID: PMCPMC46303. 8475084 10.1073/pnas.90.8.3378 PMC46303

[41] Li Y, Sun H, Ma X, Lu A, Lux R, Zusman D, et al Extracellular polysaccharides mediate pilus retraction during social motility of Myxococcus xanthus. Proc Natl Acad Sci U S A. 2003;100(9):5443–8. 10.1073/pnas.0836639100 ; PubMed Central PMCID: PMCPMC154364. 12704238 PMC154364

[42] Zhou T, Nan B. Exopolysaccharides promote Myxococcus xanthus social motility by inhibiting cellular reversals. Mol Microbiol. 2017;103(4):729–43. 10.1111/mmi.13585 . 27874229

[43] Zhuo L, Wang Y, Zhang Z, Li J, Zhang XH, Li YZ. Myxococcus xanthus DK1622 Coordinates Expressions of the Duplicate groEL and Single groES Genes for Synergistic Functions of GroELs and GroES. Front Microbiol. 2017;8:733 10.3389/fmicb.2017.00733 ; PubMed Central PMCID: PMCPMC5406781. 28496436 PMC5406781

[44] Li J, Wang Y, Zhang CY, Zhang WY, Jiang DM, Wu ZH, et al Myxococcus xanthus viability depends on groEL supplied by either of two genes, but the paralogs have different functions during heat shock, predation, and development. J Bacteriol. 2010;192(7):1875–81. 10.1128/JB.01458-09 ; PubMed Central PMCID: PMCPMC2838048. 20139189 PMC2838048

[45] Wootton JC, Drummond MH. The Q-linker: a class of interdomain sequences found in bacterial multidomain regulatory proteins. Protein Eng. 1989;2(7):535–43. . 2664763 10.1093/protein/2.7.535

[46] Stranava M, Martinek V, Man P, Fojtikova V, Kavan D, Vanek O, et al Structural characterization of the heme-based oxygen sensor, AfGcHK, its interactions with the cognate response regulator, and their combined mechanism of action in a bacterial two-component signaling system. Proteins. 2016;84(10):1375–89. 10.1002/prot.25083 . 27273553

[47] Vanatta DK, Shukla D, Lawrenz M, Pande VS. A network of molecular switches controls the activation of the two-component response regulator NtrC. Nat Commun. 2015;6:7283 10.1038/ncomms8283 . 26073186

[48] Hastings CA, Lee SY, Cho HS, Yan D, Kustu S, Wemmer DE. High-resolution solution structure of the beryllofluoride-activated NtrC receiver domain. Biochemistry. 2003;42(30):9081–90. 10.1021/bi0273866 . 12885241

[49] Kern D, Volkman BF, Luginbuhl P, Nohaile MJ, Kustu S, Wemmer DE. Structure of a transiently phosphorylated switch in bacterial signal transduction. Nature. 1999;402(6764):894–8. 10.1038/47273 . 10622255

[50] Volkman BF, Nohaile MJ, Amy NK, Kustu S, Wemmer DE. Three-dimensional solution structure of the N-terminal receiver domain of NTRC. Biochemistry. 1995;34(4):1413–24. . 7827089 10.1021/bi00004a036

[51] Fernandez I, Cornaciu I, Carrica MD, Uchikawa E, Hoffmann G, Sieira R, et al Three-Dimensional Structure of Full-Length NtrX, an Unusual Member of the NtrC Family of Response Regulators. J Mol Biol. 2017;429(8):1192–212. 10.1016/j.jmb.2016.12.022 . 28088479

[52] Lopez MF, Cabrera JJ, Salas A, Delgado MJ, Lopez-Garcia SL. Dissecting the role of NtrC and RpoN in the expression of assimilatory nitrate and nitrite reductases in Bradyrhizobium diazoefficiens. Antonie Van Leeuwenhoek. 2017;110(4):531–42. 10.1007/s10482-016-0821-3 . 28040856

[53] Skotnicka D, Smaldone GT, Petters T, Trampari E, Liang J, Kaever V, et al A Minimal Threshold of c-di-GMP Is Essential for Fruiting Body Formation and Sporulation in Myxococcus xanthus. PLoS Genet. 2016;12(5):e1006080 10.1371/journal.pgen.1006080 ; PubMed Central PMCID: PMCPMC4877007. 27214040 PMC4877007

[54] Tatke G, Kumari H, Silva-Herzog E, Ramirez L, Mathee K. Pseudomonas aeruginosa MifS-MifR Two-Component System Is Specific for alpha-Ketoglutarate Utilization. PLoS One. 2015;10(6):e0129629 10.1371/journal.pone.0129629 ; PubMed Central PMCID: PMCPMC4482717. 26114434 PMC4482717

[55] Muller S, Strack SN, Ryan SE, Shawgo M, Walling A, Harris S, et al Identification of Functions Affecting Predator-Prey Interactions between Myxococcus xanthus and Bacillus subtilis. J Bacteriol. 2016;198(24):3335–44. 10.1128/JB.00575-16 ; PubMed Central PMCID: PMCPMC5116937. 27698086 PMC5116937

[56] Taylor TB, Mulley G, Dills AH, Alsohim AS, McGuffin LJ, Studholme DJ, et al Evolution. Evolutionary resurrection of flagellar motility via rewiring of the nitrogen regulation system. Science. 2015;347(6225):1014–7. 10.1126/science.1259145 . 25722415

[57] Yang Z, Ma X, Tong L, Kaplan HB, Shimkets LJ, Shi W. Myxococcus xanthus dif genes are required for biogenesis of cell surface fibrils essential for social gliding motility. J Bacteriol. 2000;182(20):5793–8. ; PubMed Central PMCID: PMCPMC94702. 11004179 10.1128/jb.182.20.5793-5798.2000 PMC94702

[58] Volz C, Kegler C, Muller R. Enhancer binding proteins act as hetero-oligomers and link secondary metabolite production to myxococcal development, motility, and predation. Chem Biol. 2012;19(11):1447–59. 10.1016/j.chembiol.2012.09.010 . 23177199

[59] Jogler C, Waldmann J, Huang X, Jogler M, Glockner FO, Mascher T, et al Identification of proteins likely to be involved in morphogenesis, cell division, and signal transduction in Planctomycetes by comparative genomics. J Bacteriol. 2012;194(23):6419–30. 10.1128/JB.01325-12 ; PubMed Central PMCID: PMCPMC3497475. 23002222 PMC3497475

[60] Boedeker C, Schuler M, Reintjes G, Jeske O, van Teeseling MC, Jogler M, et al Determining the bacterial cell biology of Planctomycetes. Nat Commun. 2017;8:14853 10.1038/ncomms14853 ; PubMed Central PMCID: PMCPMC5394234. 28393831 PMC5394234

[61] Sharma G, Subramanian S. Unravelling the Complete Genome of Archangium gephyra DSM 2261T and Evolutionary Insights into Myxobacterial Chitinases. Genome Biol Evol. 2017;9(5):1304–11. 10.1093/gbe/evx066 ; PubMed Central PMCID: PMCPMC5441343. 28379546 PMC5441343

[62] Sharma G, Khatri I, Subramanian S. Complete Genome of the Starch-Degrading Myxobacteria Sandaracinus amylolyticus DSM 53668T. Genome Biol Evol. 2016;8(8):2520–9. 10.1093/gbe/evw151 ; PubMed Central PMCID: PMCPMC5010890. 27358428 PMC5010890

[63] Awal RP, Garcia R, Muller R. Racemicystis crocea gen. nov., sp. nov., a soil myxobacterium in the family Polyangiaceae. Int J Syst Evol Microbiol. 2016;66(6):2389–95. 10.1099/ijsem.0.001045 . 27046779

[64] Sood S, Awal RP, Wink J, Mohr KI, Rohde M, Stadler M, et al Aggregicoccus edonensis gen. nov., sp. nov., an unusually aggregating myxobacterium isolated from a soil sample. Int J Syst Evol Microbiol. 2015;65(Pt 3):745–53. 10.1099/ijs.0.061176-0 . 24591423

[65] Sharma G, Narwani T, Subramanian S. Complete Genome Sequence and Comparative Genomics of a Novel Myxobacterium Myxococcus hansupus. PLoS One. 2016;11(2):e0148593 10.1371/journal.pone.0148593 ; PubMed Central PMCID: PMCPMC4765838. 26900859 PMC4765838

[66] Kitanishi K, Kobayashi K, Uchida T, Ishimori K, Igarashi J, Shimizu T. Identification and functional and spectral characterization of a globin-coupled histidine kinase from Anaeromyxobacter sp. Fw109-5. J Biol Chem. 2011;286(41):35522–34. 10.1074/jbc.M111.274811 ; PubMed Central PMCID: PMCPMC3195594. 21852234 PMC3195594

[67] Thomas SH, Wagner RD, Arakaki AK, Skolnick J, Kirby JR, Shimkets LJ, et al The mosaic genome of Anaeromyxobacter dehalogenans strain 2CP-C suggests an aerobic common ancestor to the delta-proteobacteria. PLoS One. 2008;3(5):e2103 10.1371/journal.pone.0002103 ; PubMed Central PMCID: PMCPMC2330069. 18461135 PMC2330069

[68] Freitas TA, Saito JA, Hou S, Alam M. Globin-coupled sensors, protoglobins, and the last universal common ancestor. J Inorg Biochem. 2005;99(1):23–33. 10.1016/j.jinorgbio.2004.10.024 . 15598488

[69] Vinogradov SN, Tinajero-Trejo M, Poole RK, Hoogewijs D. Bacterial and archaeal globins—a revised perspective. Biochim Biophys Acta. 2013;1834(9):1789–800. 10.1016/j.bbapap.2013.03.021 . 23541529

[70] Hoogewijs D, Dewilde S, Vierstraete A, Moens L, Vinogradov SN. A phylogenetic analysis of the globins in fungi. PLoS One. 2012;7(2):e31856 10.1371/journal.pone.0031856 ; PubMed Central PMCID: PMCPMC3287990. 22384087 PMC3287990

[71] Pesce A, Bolognesi M, Nardini M. Protoglobin: structure and ligand-binding properties. Adv Microb Physiol. 2013;63:79–96. 10.1016/B978-0-12-407693-8.00003-0 . 24054795

[72] Burns JL, Jariwala PB, Rivera S, Fontaine BM, Briggs L, Weinert EE. Oxygen-Dependent Globin Coupled Sensor Signaling Modulates Motility and Virulence of the Plant Pathogen Pectobacterium carotovorum. ACS Chem Biol. 2017 10.1021/acschembio.7b00380 . 28612602

[73] Hou S, Larsen RW, Boudko D, Riley CW, Karatan E, Zimmer M, et al Myoglobin-like aerotaxis transducers in Archaea and Bacteria. Nature. 2000;403(6769):540–4. 10.1038/35000570 . 10676961

[74] Podgornaia AI, Laub MT. Determinants of specificity in two-component signal transduction. Curr Opin Microbiol. 2013;16(2):156–62. 10.1016/j.mib.2013.01.004 . 23352354

[75] Wojnowska M, Yan J, Sivalingam GN, Cryar A, Gor J, Thalassinos K, et al Autophosphorylation activity of a soluble hexameric histidine kinase correlates with the shift in protein conformational equilibrium. Chem Biol. 2013;20(11):1411–20. 10.1016/j.chembiol.2013.09.008 ; PubMed Central PMCID: PMCPMC3899027. 24210218 PMC3899027

[76] Adhikarla H, Wunder EA Jr., Mechaly AE, Mehta S, Wang Z, Santos L, et al Lvr, a Signaling System That Controls Global Gene Regulation and Virulence in Pathogenic Leptospira. Front Cell Infect Microbiol. 2018;8:45 Epub 2018/03/31. 10.3389/fcimb.2018.00045 ; PubMed Central PMCID: PMCPMC5863495. 29600195 PMC5863495

[77] Schug A, Weigt M, Onuchic JN, Hwa T, Szurmant H. High-resolution protein complexes from integrating genomic information with molecular simulation. Proc Natl Acad Sci U S A. 2009;106(52):22124–9. 10.1073/pnas.0912100106 ; PubMed Central PMCID: PMCPMC2799721. 20018738 PMC2799721

[78] Hwang I, Thorgeirsson T, Lee J, Kustu S, Shin YK. Physical evidence for a phosphorylation-dependent conformational change in the enhancer-binding protein NtrC. Proc Natl Acad Sci U S A. 1999;96(9):4880–5. ; PubMed Central PMCID: PMCPMC21785. 10220387 10.1073/pnas.96.9.4880 PMC21785

[79] Smith JG, Latiolais JA, Guanga GP, Pennington JD, Silversmith RE, Bourret RB. A search for amino acid substitutions that universally activate response regulators. Mol Microbiol. 2004;51(3):887–901. . 14731287 10.1046/j.1365-2958.2003.03882.x

[80] Lukat GS, Lee BH, Mottonen JM, Stock AM, Stock JB. Roles of the highly conserved aspartate and lysine residues in the response regulator of bacterial chemotaxis. J Biol Chem. 1991;266(13):8348–54. . 1902474

[81] Moore JB, Shiau SP, Reitzer LJ. Alterations of highly conserved residues in the regulatory domain of nitrogen regulator I (NtrC) of Escherichia coli. J Bacteriol. 1993;175(9):2692–701. ; PubMed Central PMCID: PMCPMC204572. 8097516 10.1128/jb.175.9.2692-2701.1993 PMC204572

[82] Pioszak AA, Ninfa AJ. Mutations altering the N-terminal receiver domain of NRI (NtrC) That prevent dephosphorylation by the NRII-PII complex in Escherichia coli. J Bacteriol. 2004;186(17):5730–40. 10.1128/JB.186.17.5730-5740.2004 ; PubMed Central PMCID: PMCPMC516846. 15317778 PMC516846

[83] Vance RE, Zhu J, Mekalanos JJ. A constitutively active variant of the quorum-sensing regulator LuxO affects protease production and biofilm formation in Vibrio cholerae. Infect Immun. 2003;71(5):2571–6. 10.1128/IAI.71.5.2571-2576.2003 ; PubMed Central PMCID: PMCPMC153284. 12704130 PMC153284

[84] Boyaci H, Shah T, Hurley A, Kokona B, Li Z, Ventocilla C, et al Structure, Regulation, and Inhibition of the Quorum-Sensing Signal Integrator LuxO. PLoS Biol. 2016;14(5):e1002464 10.1371/journal.pbio.1002464 ; PubMed Central PMCID: PMCPMC4878744. 27219477 PMC4878744

[85] Bretscher AP, Kaiser D. Nutrition of Myxococcus xanthus, a fruiting myxobacterium. J Bacteriol. 1978;133(2):763–8. ; PubMed Central PMCID: PMCPMC222085. 415048 10.1128/jb.133.2.763-768.1978 PMC222085

[86] Muller S, Willett JW, Bahr SM, Darnell CL, Hummels KR, Dong CK, et al Draft Genome Sequence of Myxococcus xanthus Wild-Type Strain DZ2, a Model Organism for Predation and Development. Genome Announc. 2013;1(3). 10.1128/genomeA.00217-13 ; PubMed Central PMCID: PMCPMC3650445. 23661486 PMC3650445

[87] Ulrich LE, Zhulin IB. The MiST2 database: a comprehensive genomics resource on microbial signal transduction. Nucleic Acids Res. 2010;38(Database issue):D401–7. 10.1093/nar/gkp940 ; PubMed Central PMCID: PMCPMC2808908. 19900966 PMC2808908

[88] Wu SS, Kaiser D. Markerless deletions of pil genes in Myxococcus xanthus generated by counterselection with the Bacillus subtilis sacB gene. J Bacteriol. 1996;178(19):5817–21. ; PubMed Central PMCID: PMCPMC178429. 8824635 10.1128/jb.178.19.5817-5821.1996 PMC178429

[89] Ueki T, Inouye S, Inouye M. Positive-negative KG cassettes for construction of multi-gene deletions using a single drug marker. Gene. 1996;183(1–2):153–7. . 8996101 10.1016/s0378-1119(96)00546-x

[90] Kashefi K, Hartzell PL. Genetic suppression and phenotypic masking of a Myxococcus xanthus frzF- defect. Mol Microbiol. 1995;15(3):483–94. . 7783619 10.1111/j.1365-2958.1995.tb02262.x

[91] Julien B, Kaiser AD, Garza A. Spatial control of cell differentiation in Myxococcus xanthus. Proc Natl Acad Sci U S A. 2000;97(16):9098–103. ; PubMed Central PMCID: PMCPMC16828. 10922065 10.1073/pnas.97.16.9098 PMC16828

[92] Black WP, Yang Z. Myxococcus xanthus chemotaxis homologs DifD and DifG negatively regulate fibril polysaccharide production. J Bacteriol. 2004;186(4):1001–8. 10.1128/JB.186.4.1001-1008.2004 ; PubMed Central PMCID: PMCPMC344214. 14761994 PMC344214

[93] Finn RD, Coggill P, Eberhardt RY, Eddy SR, Mistry J, Mitchell AL, et al The Pfam protein families database: towards a more sustainable future. Nucleic Acids Res. 2016;44(D1):D279–85. 10.1093/nar/gkv1344 ; PubMed Central PMCID: PMCPMC4702930. 26673716 PMC4702930

[94] Oberto J. SyntTax: a web server linking synteny to prokaryotic taxonomy. BMC Bioinformatics. 2013;14:4 10.1186/1471-2105-14-4 ; PubMed Central PMCID: PMCPMC3571937. 23323735 PMC3571937

[95] Bienert S, Waterhouse A, de Beer TA, Tauriello G, Studer G, Bordoli L, et al The SWISS-MODEL Repository-new features and functionality. Nucleic Acids Res. 2017;45(D1):D313–D9. 10.1093/nar/gkw1132 ; PubMed Central PMCID: PMCPMC5210589. 27899672 PMC5210589

[96] Willett JW, Kirby JR. Genetic and biochemical dissection of a HisKA domain identifies residues required exclusively for kinase and phosphatase activities. PLoS Genet. 2012;8(11):e1003084 10.1371/journal.pgen.1003084 ; PubMed Central PMCID: PMCPMC3510030. 23226719 PMC3510030

