# Supplementary material for: Suppressor mutations reveal an NtrC-like response regulator, NmpR, for modulation of Type-IV Pili-dependent motility in Myxococcus xanthus
Source: PLoS Genet. 2018 Oct 22;14(10):e1007714. doi: 10.1371/journal.pgen.1007714 (PMC6211767; doi:10.1371/journal.pgen.1007714)
Supplement: S1 Table — (DOCX) [file pgen.1007714.s001.docx]

S1 Table. Bacterial Strains and Plasmids

| **Strain** | **Relevant genotype** | **Source** |
| --- | --- | --- |
| *M. xanthus* |  |  |
| DZ2 | Wild-type | [1] |
| JK4281 | Δ*pilR* | [2] |
| JK4456 | C1 Δ*pilR* suppressor | This study |
| JK4467 | C2; Δ*pilR* suppressor *mxan_4246* C181T | This study |
| JK4462 | C3; Δ*pilR* suppressor *mxan_4246* C649T | This study |
| JK4459 | C4; Δ*pilR* suppressor *mxan_4246* C649T | This study |
| JK4460 | C5; Δ*pilR* suppressor *mxan_4246* C847T | This study |
| JK4455 | C6; Δ*pilR* suppressor *mxan_4246* Δ-1(1085) | This study |
| JK4464 | C7; Δ*pilR* suppressor *mxan_4246* Δ-1(1104) | This study |
| JK4457 | C8; Δ*pilR* suppressor *mxan_4246* Δ-1(1104) | This study |
| JK4461 | C9; Δ*pilR* suppressor *mxan_4240* T260 | This study |
| JK4675 | *M. xanthus* DZ2 Δ*mxan_4240* | This study |
| JK4907 | *M. xanthus* DZ2 Δ*mxan_4246* | This study |
| JK4716 | Δ*pilR* Δ*mxan_4240* | This study |
| JK4682 | Δ*pilR* Δ*mxan_4246* | This study |
| JK4849 | Δ*pilR* Δ*mxan_4246* Δ*mxan_4240* | This study |
| JK4953 | Δ*pilR* Δ*mxan_4246* Δ*mxan_4244* | This study |
| JK4718 | JK4467 (C2) Δ*mxan_4240* | This study |
| JK4680 | JK4467 (C2) Δ*mxan_4246* | This study |
| JK4776 | JK4459 (C4) Δ*mxan_4240* | This study |
| JK4786 | JK4459 (C4) Δ*mxan_4246* | This study |
| JK4770 | JK4460 (C5) Δ*mxan_4240* | This study |
| JK4685 | JK4460 (C5) Δ*mxan_4246* | This study |
| JK4949 | JK4460 (C5) Δ*mxan_4244* | This study |
| JK4676 | JK4461 (C9) Δ*mxan_4240* | This study |
| JK4772 | JK4461 (C9) Δ*mxan_4246* | This study |
| JK4834 | JK4770 (C5) Δ*mxan_4240* with pNat-4240 | This study |
| JK4793 | JK4770 (C5) Δ*mxan_4240* with pHigh-4240 | This study |
| JK4838 | JK4770 (C5) Δ*mxan_4240* with pHigh-4240D54A | This study |
| JK4836 | JK4770 (C5) Δ*mxan_4240* with pHigh-4240D54E | This study |
| JK4832 | JK4676 (C9) Δ*mxan_4240* with pNat-4240 | This study |
| JK4787 | JK4676 (C9) Δ*mxan_4240* with pHigh-4240 | This study |
| JK4816 | JK4676 (C9) Δ*mxan_4240* with pHigh-4240D54A | This study |
| JK4817 | JK4676 (C9) Δ*mxan_4240* with pHigh-4240D54E | This study |
| JK4846 | JK4676 (C9) Δ*mxan_4240* with pNat-4240V87E | This study |
| JK4662 | *M. xanthus* DZ2 with pCD127-*pGroES* | This study |
| JK4811 | Δ*pilR* with pCD127-*pGroES* | This study |
| JK4905 | JK4461 (C9) with pCD127-*pGroES* | This study |
| JK4702 | Δ*pilR* with pHigh-4240 | This study |
| JK5025 | *M. xanthus* DZ2 with pHigh-NmpS | This study |
| JK5082 | *M. xanthus* DZ2 with pHigh-NmpS D59A | This study |
| JK5027 | Δ*pilR* with pHigh-NmpS | This study |
| JK5086 | Δ*pilR* with pHigh-NmpS D59A | This study |
| JK5104 | JK4460 (C5) Δ*pilS* | This study |
| JK5102 | JK4461 (C9) Δ*pilS* | This study |
|  |  |  |
| **Plasmid** | **Use** | **Source** |
| pCR2.1-TOPO | Cloning Vector | Invitrogen |
| pBJ113-4240 | Gene deletion; *mxan_4240* | This study |
| pBJ113-4246 | Gene deletion; *mxan_4246* | This study |
| pBJ113-4244 | Gene deletion; *mxan_4244* | This study |
| pSUM117 | Complementation | This study |
| pSUM117-pNat | Complementation; 585 bp upstream of mxan_4236 | This study |
| pSUM117-pHigh | Complementation; 623 bp upstream of *mxan_4894*, *groES* | This study |
| pSUM117-pNat4240 | Complementation; wild-type Mxan_4240 | This study |
| pSUM117-pNat4240V87E | Complementation; Mxan_4240 V87E | This study |
| pSUM117-pHigh4240 | Complementation; wild-type Mxan_4240 | This study |
| pSUM117-pHigh4240D54A | Complementation; Mxan_4240 D54A | This study |
| pSUM117-pHigh4240D54E | Complementation; Mxan_4240 D54E | This study |
| pSUM117-pHigh4240V87E | Complementation; Mxan_4240 V87E | This study |
| pCD127 | *lacZ* expression | [3] |
| pCD127-p*GroES* | 623 bp upstream of *mxan_4894*, *groES*; *lacZ* expression | This study |
| pET28a-4240Rec | Receiver domain of NmpR | This study |
| pET28a-4244HK | Kinase domain of NmpS | This study |
| pET28a-4244Rec | Receiver domain of NmpS | This study |
| pET28a-4245Rec1 | First receiver domain of NmpT | This study |
| pET28a-4245Rec2 | Second receiver domain of NmpT | This study |
| pET28a-4246HK | Kinase domain of NmpU | This study |
| pET28a-NmpS | Full-length NmpS | This study |
| pET28a-NmpSD59A | Full-length NmpS with a D59A substitution | This study |
| pET28a-NmpR | Full-length NmpR | This study |
