# Supplementary material for: Suppressor mutations reveal an NtrC-like response regulator, NmpR, for modulation of Type-IV Pili-dependent motility in Myxococcus xanthus
Source: PLoS Genet. 2018 Oct 22;14(10):e1007714. doi: 10.1371/journal.pgen.1007714 (PMC6211767; doi:10.1371/journal.pgen.1007714)
Supplement: S2 Table — (DOCX) [file pgen.1007714.s002.docx]

S2 Table. Primers used in this study

| **Primer** | **Sequence** | **Description** |
| --- | --- | --- |
| 4240upF-EcoRI | AAGAATTCCATGCCCTTCGTCATCAGC | Gene deletion |
| 4240upR-XbaI | AATCTAGAGGGGGAGGTGGAGCCTTT | Gene deletion |
| 4240downF-XbaI | AATCTAGACGGTTTGATGGGTGGGCT | Gene deletion |
| 4240downR-HindIII | AAAAGCTTAGCACGGCCTTGAAGCG | Gene deletion |
| 4246upF-EcoRI | AAGAATTCGGGCACGCTTCAAGTCCG | Gene deletion |
| 4246upR-XbaI | AATCTAGAGAGTCCTGGGGTAAGAGCCTATCC | Gene deletion |
| 4246downF-XbaI | AATCTAGAGCTTCGTGATTCGGCTTTCC | Gene deletion |
| 4246downR-HindIII | AAAAGCTTCTCGCGTGGCGTGACTTC | Gene deletion |
| 4244downF-xbaI | AATCTAGAGGCCCGTCAGCGGAATGTAG | Gene deletion |
| 4244downR-HindIII | AAAGCTTCGAAGGCGGAGCGAGCAC | Gene deletion |
| 4244upF-EcoRI | AAGAATTCGTGATTCGGCTTTCCCTCGTC | Gene deletion |
| 4244upR-XbaI | AATCTAGATCATAAGGCGTGGGTGGGAC | Gene deletion |
| mxan_4240conf-fwd | GTCCGCCTCATCTTCACCGAG | Confirm PCR |
| mxan_4240conf-rev | CATATTGGCGCTGAGACCAGACG | Confirm PCR |
| mxan_4240conf-inter | GTCCAACGGCTTGAGCAGGAAG | Confirm PCR |
| mxan_4246conf-fwd | CATTCGCGGTGCGCCACAGG | Confirm PCR |
| mxan_4246conf-rev | GAGGTGGTGCACGGCATCCG | Confirm PCR |
| mxan_4246conf-inter | CATCGCGCCGAACATGTAGTGC | Confirm PCR |
| hsp60conf-fwd | AGGTGCGAGTGCTAACCCCAT | Confirm PCR |
| 4240conf-fwd-2 | GACCAGCCGCCGCATCTCA | Confirm PCR |
| 4240conf-rev-2 | GTTGTTCGCAGAAGAAGTCCACCTG | Confirm PCR |
| 4244conf-F | GACCACGGGCGTCTTCTCC | Confirm PCR |
| 4244conf-R | CTACGTATTCGACGTCTGGTTCAC | Confirm PCR |
| 4240V87Efwdquick | GTTCGCCAGCGAGGAGACGGCCGTG | Mutagenesis |
| 4240V87Erevquick | CACGGCCGTCTCCTCGCTGGCGAAC | Mutagenesis |
| 4240D54A-fwd | GGTGCTCACCGCCGTGCGCATGCCG | Mutagenesis |
| 4240D54A-rev | CGGCATGCGCACGGCGGTGAGCACC | Mutagenesis |
| 4240D54E-fwd | GGTGCTCACCGAAGTGCGCATGCCG | Mutagenesis |
| 4240D54E-rev | CGGCATGCGCACTTCGGTGAGCACC | Mutagenesis |
| 4236proF-SacI | AAGAGCTCCTGGAGTCGTAGACGTGCACC | Complementation |
| 4236proR-XbaI | AATCTAGACATGGCGGTACTCCTGGCA | Complementation |
| pHsp60F-XbaI | AATCTAGACTGGTGGTAGAGGATGGCCAG | Complementation |
| pHsp60R-HindIII | AAAAGCTTCATGGCCTGCTCCTTACATGTC | Complementation |
| 4246F-XbaI | AATCTAGAATGGCGGAAACCTTGTTCGA | Complementation |
| 4246R-HindIII | AAAAGCTTTCATCCAACAGGAGGTACTGGC | Complementation |
| 4240F-XbaI | AATCTAGAATGTCCCCACAGCGAATCCT | Complementation |
| 4240R-HindIII | AAAAGCTTCTACGACTCCGCGGCCAG | Complementation |
| 4240RecFwdNdeI | AAACATATGTCCCCACAGCGAATCCTG | Protein Purification |
| 4240RecRevHindIII | AAAAAGCTTTCACTTCTCCAGCACCTTGCC | Protein Purification |
| 4240-FullRev-HindIII | AAAAAGCTTCTACGACTCCGCGGCCAG | Protein Purification |
| 4246HKFwdNdeI | AAACATATGGGCCAACTGGTGGGTTCC | Protein Purification |
| 4246HKRevHindIII | AAAAAGCTTTCATCCAACAGGAGGTACTGGC | Protein Purification |
| 4245Rec1FwdNdeI | AAACATATGCGCCAGTACCTCCTGTTGGAT | Protein Purification |
| 4245Rec1RevHindIII | AAAAAGCTTTCAATCCCGGCGAGCACC | Protein Purification |
| 4245Rec2FwdNdeI | AAACATATGGGGTTGGTGGTGCTGGTG | Protein Purification |
| 4245Rec2RevHindIII | AAAAAGCTTTCATAAGGCGTGGGTGGGAC | Protein Purification |
| 4244RecFwdNdeI | AAACATATGAGCGAGCCCACCTCTCCG | Protein Purification |
| 4244RecRevHindIII | AAAAAGCTTTCACTGCCGCATCGCTTGCTC | Protein Purification |
| 4244HKFwdNdeI | AAACATATGGGGACGATGACGGCGG | Protein Purification |
| 4244HKRevHindIII | AAAAAGCTTCTACCCCGCCAGCGGC | Protein Purification |
| Pil Probe 1 fwd | CTCCTCGCAGTGAATTGGTGG | EMSA |
| Pil Probe 1 rev | TCACAGCGGCAACCCC | EMSA |
| Pil Probe 2 fwd | GTTCGCGGCTCGTGTGG | EMSA |
| Pil Probe 2 rev | CAGGATGCGCCGCTTC | EMSA |
| Pil Probe 3 fwd | GACCTACCCGATCATCGTGATC | EMSA |
| Pil Probe 3 rev | GAGGCTCGCGGCGAC | EMSA |
| Pil Probe 4 fwd | GCTGCTGCTGCGGCG | EMSA |
| Pil Probe 4 rev | GCGTCACCTTCAGCAGCAG | EMSA |
| Pil Probe 5 fwd | CGTGAAGGGCCTGTCGC | EMSA |
| Pil Probe 5 rev | TAGAGCGTCCGAAGGCCTCG | EMSA |
| Pil Probe 6 fwd | CACGGGCCACCTGGTG | EMSA |
| Pil Probe 6 rev | CGCCACGCTGCATGAAG | EMSA |
| Pil Probe 7 fwd | CATCGAGGTCACCAAGGAAGAGAC | EMSA |
| Pil Probe 7 rev | TGCCCGGGGCGTC | EMSA |
| Pil Probe 8 fwd | GCAATTTTCCCGTGTCCGTT | EMSA |
| Pil Probe 8 rev | CCTTGGCCATCTCATCGATGTT | EMSA |
| Pil Probe 9a rev | GTGGATCCACCCGGAGCA | EMSA |
| Pil Probe 9b fwd | TGGCACGTGACGTACGGG | EMSA |
| Pil Probe 10 Fwd | GCTCGAACCGTGATGAGGG | EMSA |
| Pil Probe 10 Rev | CCTCGTCCAACATGACGGTG | EMSA |
| Pil Probe 11 Fwd | CTCTGGCCTCCTGGTGGC | EMSA |
| Pil Probe 11 Rev | CTACGCATCCGAAGCGCTC | EMSA |

**Supplemental Table References**

1. Muller S, Willett JW, Bahr SM, Darnell CL, Hummels KR, Dong CK, et al. Draft Genome Sequence of Myxococcus xanthus Wild-Type Strain DZ2, a Model Organism for Predation and Development. Genome Announc. 2013;1(3). doi: 10.1128/genomeA.00217-13. PubMed PMID: 23661486; PubMed Central PMCID: PMCPMC3650445.

2. Bretl DJ, Muller S, Ladd KM, Atkinson SN, Kirby JR. Type IV-pili dependent motility is co-regulated by PilSR and PilS2R2 two-component systems via distinct pathways in Myxococcus xanthus. Mol Microbiol. 2016;102(1):37-53. doi: 10.1111/mmi.13445. PubMed PMID: 27393239.

3. Darnell CL, Wilson JM, Tiwari N, Fuentes EJ, Kirby JR. Chemosensory regulation of a HEAT-repeat protein couples aggregation and sporulation in Myxococcus xanthus. J Bacteriol. 2014;196(17):3160-8. doi: 10.1128/JB.01866-14. PubMed PMID: 24957622; PubMed Central PMCID: PMCPMC4135651.
