# Supplementary figures and images for: Suppressor mutations reveal an NtrC-like response regulator, NmpR, for modulation of Type-IV Pili-dependent motility in Myxococcus xanthus
Source: PLoS Genet. 2018 Oct 22;14(10):e1007714. doi: 10.1371/journal.pgen.1007714 (PMC6211767; doi:10.1371/journal.pgen.1007714)

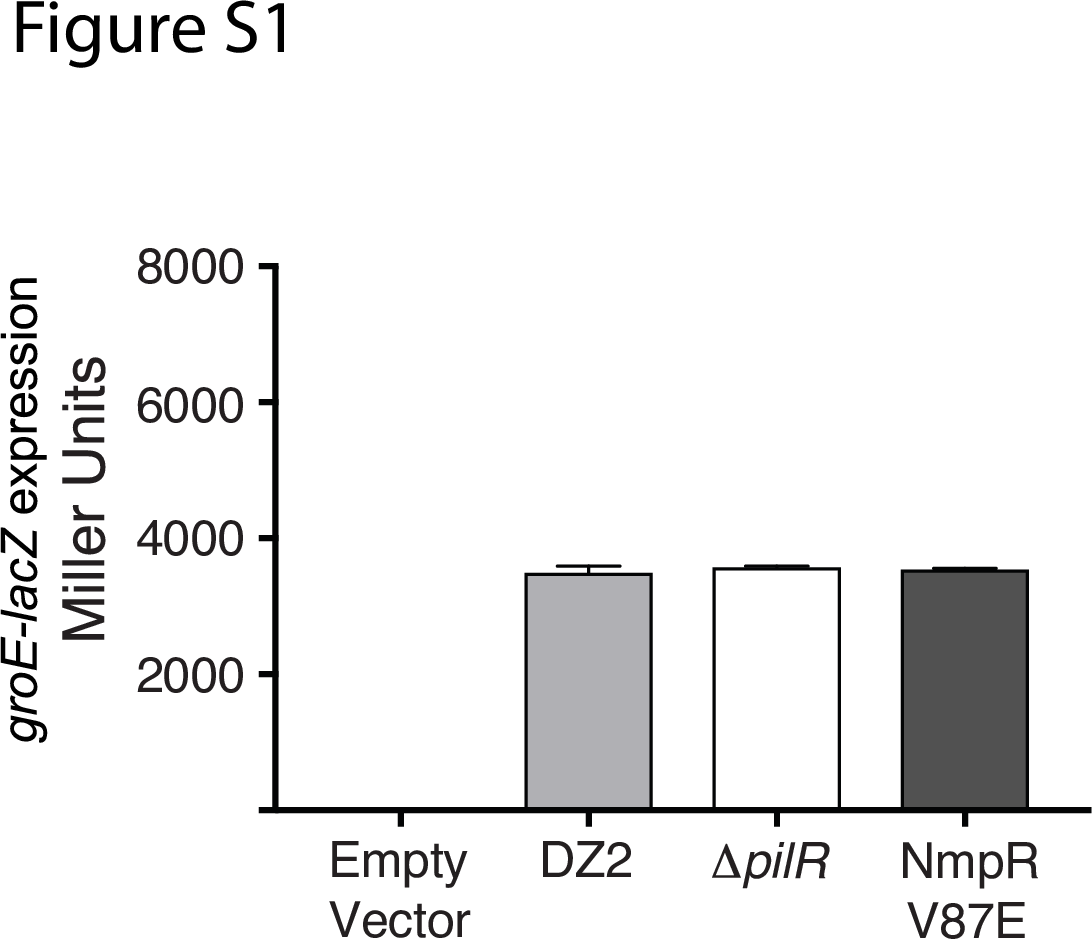

Supplement: S1 Fig — To determine if the groES promoter was reasonable to use for complementation experiments, a groES-lacZ reporter was constructed and transformed into relevant strains used in this research. The Miller Units reported in this Figure are directly comparable to the levels of the pilA reporter: ((OD420/mg protein * time in minutes)*1000). The levels of groES expression are essentially identical between the strains tested here, when assayed following growth on a hard agar surface. (TIFF) [file pgen.1007714.s003.tiff]

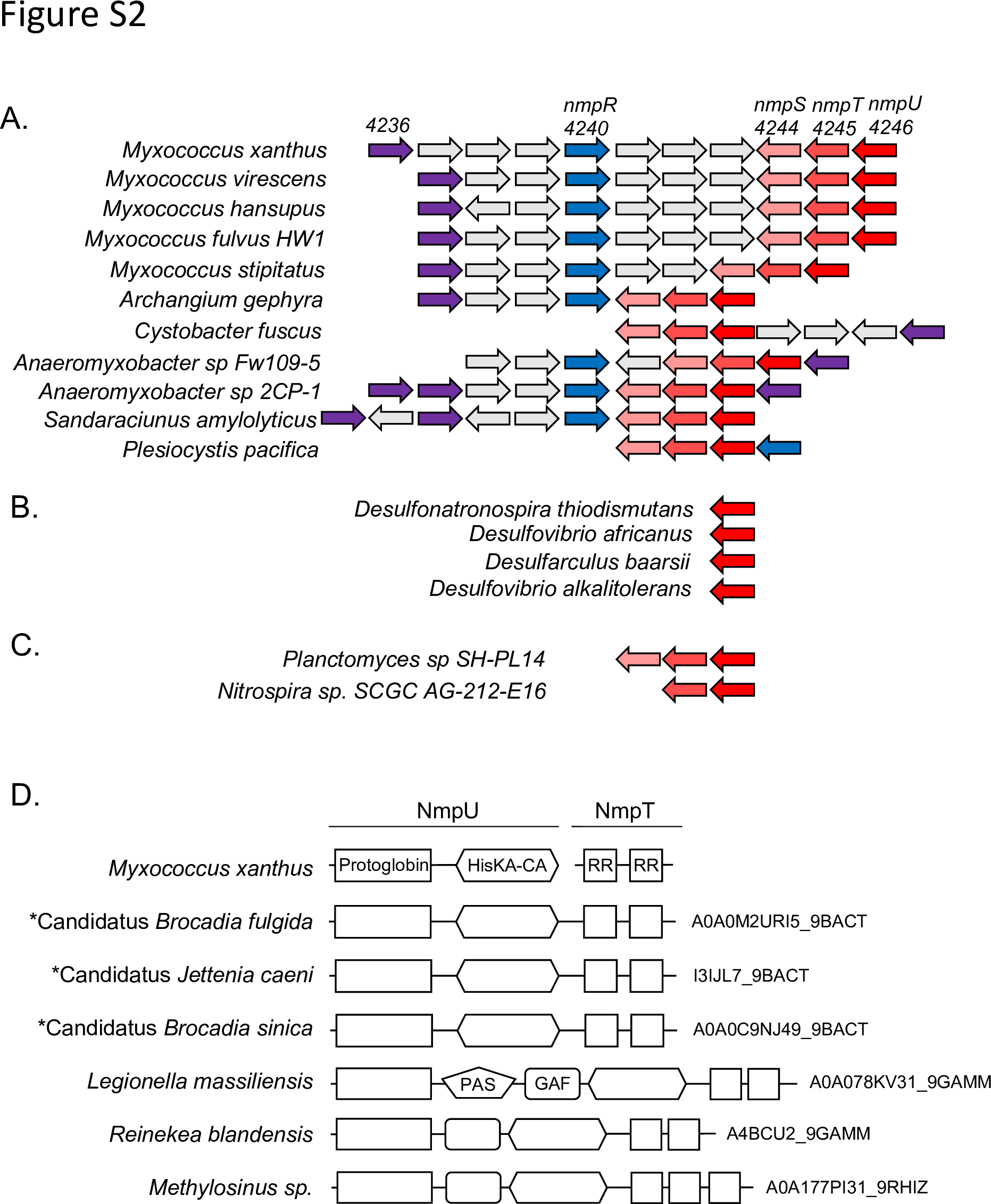

Supplement: S2 Fig — Some members of the ∂-proteobacteria contain homologues of the multi-component signaling pathway (NmpR = blue, NmpSTU in shades of red) (A). Mxan_4236 (purple) is a CBS domain protein and serves as a convenient landmark of the genomic location (A). Only a NmpU homologue could be identified in more distantly related ∂-proteobacteria (B). Shared synteny was also identified outside of the ∂-proteobacteria (C) and multiple examples of NmpU/NmpT homologues (D) were identified outside of the ∂-proteobacteria. Those species identified with a * are all members of the Planctomycetes phylum. Collectively, these observations suggest these genes have been exchanged by horizontal gene transfer followed by divergent evolution of domain architectures. (TIFF) [file pgen.1007714.s004.tiff]
